# Neoleukin-2/15-armored CAR-NK cells sustain superior therapeutic efficacy in solid tumors via c-Myc/NRF1 activation

**DOI:** 10.1038/s41392-025-02158-2

**Published:** 2025-03-03

**Authors:** Jianhua Luo, Meng Guo, Mingyan Huang, Yanfang Liu, Yuping Qian, Qiuyan Liu, Xuetao Cao

**Affiliations:** 1 National Key Laboratory of Immunity & Inflammation, Institute of Immunology, Navy Medical University, Shanghai, 200433 China; 2 Department of Pathology, Changhai Hospital, Navy Medical University, Shanghai, 200433 China; 3 Department of Immunology, Center for Immunotherapy, Institute of Basic Medical Sciences, Peking Union Medical College, Chinese Academy of Medical Sciences, Beijing, 100005 China; 4 Institute of Immunology, College of Life Sciences, Nankai University, Tianjin, 300071 China

**Keywords:** Tumour immunology, Molecular medicine

## Abstract

Adoptive transfer of chimeric antigen receptor (CAR)-modified natural killer (NK) cells represents a transformative approach that has significantly advanced clinical outcomes in patients with malignant hematological conditions. However, the efficacy of CAR-NK cells in treating solid tumors is limited by their exhaustion, impaired infiltration and poor persistence in the immunosuppressive tumor microenvironment (TME). As NK cell functional states are associated with IL-2 cascade, we engineered mesothelin-specific CAR-NK cells that secrete neoleukin-2/15 (Neo-2/15), an IL-2Rβγ agonist, to resist immunosuppressive polarization within TME. The adoptively transferred Neo-2/15-armored CAR-NK cells exhibited enhanced cytotoxicity, less exhaustion and longer persistence within TME, thereby having superior antitumor activity against pancreatic cancer and ovarian cancer. Mechanistically, Neo-2/15 provided sustained and enhanced downstream IL-2 receptor signaling, which promotes the expression of c-Myc and nuclear respiratory factor 1 (NRF1) in CAR-NK cells. This upregulation was crucial for maintaining mitochondrial adaptability and metabolic resilience, ultimately leading to increased cytotoxicity and pronounced persistence of CAR-NK cells within the TME. The resistance against TME immunosuppressive polarization necessitated the upregulation of NRF1, which is essential to the augmentative effects elicited by Neo-2/15. Overexpression of NRF1 significantly bolsters the antitumor efficacy of CAR-NK cells both in vitro and in vivo, with increased ATP production. Collectively, Neo-2/15-expressing CAR-NK cells exerts superior antitumor effects by exhaustion-resistance and longer survival in solid tumors.

## Introduction

One of the most promising applications in cancer immunotherapy lies in the adoptive transfer of chimeric antigen receptor (CAR)-engineered immune cells.^1^ CAR is a sophisticatedly designed fusion protein that consists of an extracellular domain capable of specifically recognizing a tumor antigen, and tandem intracellular domains that activate immune responses.^2,3^ T cells were the first adoptive cell type to be engineered with CAR segments, known as CAR-T.^3^ While this approach has achieved significant successes in treating several types of cancers, its broader application has been constrained by a high incidence of cytokine release syndrome (CRS) and potential severe immunotoxicity.^3^ Natural killer (NK) cells, with cytotoxic functions similar to those of CD8^+^ T cells, represent a viable alternative due to their distinct cytokine profile which minimize the risk of CRS. Clinical trials involving extensive allogeneic NK cell transplants have further demonstrated their low risk for graft-versus-host disease (GvHD).^4^ Consequently, NK cells have emerged as an excellent candidate for CAR engineering, offering a safer profile for adoptive cell therapy in the context of broader clinical applications.

At present, CAR-NK cell therapy has demonstrated significant efficacy in treating hematologic malignancies.^5,6^ However, the available clinical evidence regarding the efficacy of CAR-NK cells in the treatment of solid tumors, particularly pancreatic ductal adenocarcinoma (PDAC), remains scarce.^7^ Mechanistically, the efficacy of CAR-NK cells in treating PDAC is limited due to the immunologically quiescent tumor microenvironment (TME), characterized by an abundance of stromal cells and extracellular matrix but a lack of vascularization, resulting in a harsh metabolic landscape.^8^ In the presence of hypoxia, cancer cells demonstrate a notable level of metabolic adaptability, which is evidenced by the heightened expression of nutrient transporters and the utilization of the micropinocytosis pathway to acquire necessary nutrients for sustaining their energy demands.^9^ The deprivation of essential amino acids and glucose in TME can potentially lead to the senescence of adoptive immune cells and the exhaustion of endogenous immunity.^10,11^ The immunosuppressive features of the TME, including a deficiency in immunostimulatory cytokines, enable solid tumor cells to evade CAR-NK cell-mediated tumor clearance by impairing their cytotoxicity and reducing their persistence.^12,13^ As a result, CAR-NK cells that have been able to infiltrate solid tumors are dysfunctional, exhausted and metabolically impaired.^14^ Interventions that resist NK cell immunosuppressive polarization following adoptive transfer have the potential to overcome these deficiencies and improve the efficacy of CAR-NK cell therapy of solid tumors.

To overcome the detrimental effects of TME-caused immunosuppression, various strategies have been utilized to augment the therapeutic efficacy of CAR-engineered immune cells, including the upregulation of chemokine receptors to enhance cell infiltration into tumor tissue,^15,16^ disrupting the cancer-associated fibroblasts with anti-fibroblast activating protein CAR,^17^ as well as the depletion of transcription factors associated with adoptive cell exhaustion.^18^ However, these approaches have yet to address the challenges pertaining to the energy supply and survival of adoptively transferred immune cells infiltrated within TME. Mitochondria are vital organelles that are involved in energy metabolism through tricarboxylic acid (TCA) cycle and oxidative phosphorylation (OXPHOS),^19,20^ and the metabolic resilience of NK cells, which is closely tied to their effector functions, can be modulated by cytokine signaling.^21^ In particular, the interleukin (IL)-2 or IL-15 is pivotal in augmenting NK cell metabolism and function through the activation of the JAK-STAT, MAPK, and PI3K-Akt-mTOR signaling pathways, which collectively enhance glycolysis and OXPHOS.^12^ Given the metabolic challenges faced by NK cells within TME, the strategies that engineer NK cells to respond robustly to IL-2/IL-15 signaling may potentiate their antitumor responses.

The IL-2 and IL-15 receptors both incorporate the shared β subunit (IL-2Rβ) and the common gamma chain (γc), while also pairing with their distinct third subunit, IL-2Rα or IL-15Rα, respectively.^22^ The adverse effects mediated by natural IL-2 and IL-15 are primarily attributed to their binding activity through their respective α chains.^23,24^ Hence, the design of “superkines” like H9, H9T, N72D, and neoleukin-2/15 (Neo-2/15), which act as IL-2Rβγ biased agonists, is envisaged to facilitate lymphocyte activation and circumvent the collateral effects.^25–28^ Considering that the cytokine-armoring approaches have been recently shown to boost the antitumor capacity of adoptive cells, we wanted to determine the most appropriate IL-2R ligand in promoting CAR-NK cell proliferation, perforin (PFN) and granzyme B (GrB) secretion, as well as the cytotoxicity. Therefore, the primary aim of our investigation is to specifically address the potential of autocrine superkines in CAR-NK cell therapy for solid tumors, an area that, to our knowledge, has not been extensively studied to date. In this study, we demonstrate that Neo-2/15-armored mesothelin (MSLN)-specific CAR-NK cells exert superior antitumor effects by exhaustion-resistance and longer survival in solid tumors. This enhancement in CAR-NK effector function is due to the activation of the IL-2Rβγ signaling cascade, which subsequently activates STAT5/Akt pathway and downstream c-Myc/nuclear respiratory factor 1 (NRF1) signaling, leading to the increased NK cell resistance to immunosuppressive polarization in TME. Moreover, the overexpression of NRF1 improved the antitumor effectiveness of CAR-NK cells alongside enhanced ATP generation. Our findings suggest that optimizing the metabolism of therapeutic NK cells could significantly invigorate the antitumor efficacy of engineered NK cells.

## Results

### Neo-2/15 potently enhances activation but prevents exhaustion of NK cells

**Fig. 1:**
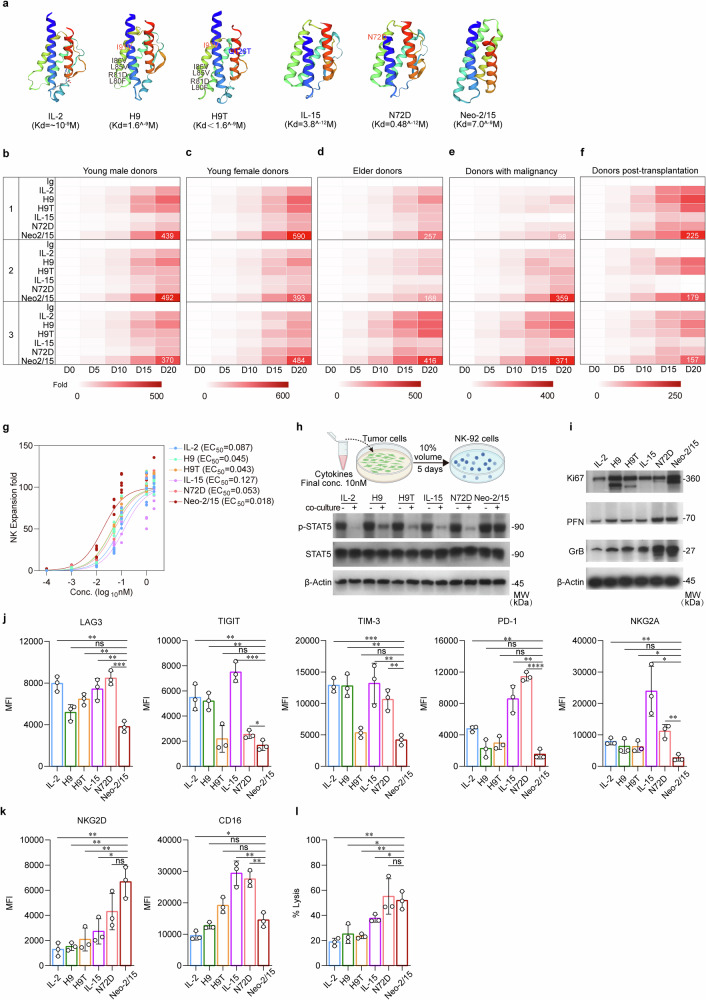
Neo-2/15 is the optimal IL-2R agonist for NK cells. **a** Protein structures of natural human IL-2 and IL-15 as well as superkines H9, H9T, N72D and Neo-2/15 and their binding affinities to IL-2Rβγ predicted using the Swisse model. Kd: equilibrium binding dissociation value. **b** Heatmap showing the expansion of human primary NK cells obtained from healthy young males (age < 34 years) at indicated days after being stimulated with 1 nM indicated cytokine or superkine. The number 1, 2, and 3 indicate three different individuals for each group. **c** Heatmap of the expansion of primary NK cells from healthy young females (age < 41 years). **d** Heatmap of the expansion of primary NK cells from healthy elder individuals (age > 60 years). **e** Heatmap of the expansion of primary NK cells from cancer patients. **f** Heatmap of the expansion of primary NK cells from organ transplant recipients receiving immunosuppressants (FK506 plus Mycophenolate Mofetil). **b**-**f** *n* = 6. **g** The proliferation of NK-92 incubated with indicated cytokine or superkine for 1 week is quantified by cell expansion fold. Data were fitted with a logistic equation (smooth curves) to estimate the EC_50_ value. **h** Schematic representation of testing the stability of IL-2R ligands in vitro (top). Different cytokine or superkine is individually added to the medium of AsPC-1 cell cultures, the culture supernatant is collected 5 days later and added to cytokine-free NK-92 culture medium at 1:10 ratio. NK-92 are harvested 1 hour later, then the total and the phosphorylated STAT5 in cell lysates are detected by immunoblotting (bottom). -: complete NK-92 culture medium with 1 nM indicated cytokine or supekine. +: cytokine-free NK-92 culture medium with 10% tumor-culture supernatant contained indicated cytokine or supekine. β-Actin served as loading control. **i** NK-92 stimulated with 1 nM indicated cytokine or superkine for 4 hours are subjected to immunoblotting using indicated antibodies. β-Actin served as the loading control. The expression of LAG3, TIGIT, TIM-3, PD-1, NKG2A (**j**), NKG2D and CD16 (**k**) in primary NK cells derived from healthy donors stimulated with 1 nM indicated cytokine or superkine for 4 hours were detected by flow cytometry. Data are presented as mean ± SD (ns, not significant, *P < 0.05, **P < 0.01, ***P < 0.001, *n* = 3). **l** Cytotoxicity of IL-2, IL-15, H9, H9T, N72D or Neo-2/15 stimulated human primary NK cells obtained from healthy young males (age < 35 years) on K562 target cells after co-culture for 4 hours at E:T ratios of 1:1 was detected by flow cytometry (ns, not significant, *P < 0.05, **P < 0.01, *n* = 3)

IL-2 and IL-15 share the IL-2Rβγ receptor subunits. In order to provide a strong basis for promoting NK cell proliferation and activity, we screened these natural cytokines along with their analog superkines H9, H9T, N72D, and Neo-2/15 to identify the optimal IL-2R stimulant for enhancing NK cell functions (Fig. 1a). We first analyzed their effects on the proliferation of NK cells derived from the peripheral blood of young and elder healthy individuals, cancer patients receiving chemotherapy, and organ transplant recipients receiving immunosuppressants. Among these agonists, Neo-2/15 most significantly enhanced the expansion of primary NK cells across multiple donors (Fig. 1b–f and Supplementary Fig. 1a–e). Neo-2/15 also most significantly enhanced the proliferation of the human NK cell line NK-92, with an EC_50_ value of 0.018 nM compared to 0.087 nM for IL-2 or 0.043 nM for H9T (Fig. 1g).

To evaluate the stability of these agonists, they were added into the culture medium of the human pancreatic cancer cell line AsPC-1, and the supernatant was collected 5 days later and used to treat NK-92 (Fig. 1h, top). The culture media containing the agonist could all induce phosphorylation of STAT5, however, when cultured in the presence of tumor cell supernatant, only Neo-2/15 could maintain STAT5 phosphorylation compared to other agonists in NK-92 (Fig. 1h, bottom) with less degradation (Supplementary Fig. 1f), suggesting high stability of Neo-2/15 within the supernatant. Given that AsPC-1 is a high HMGB1-expressing cell line and HMGB1 can activate NK cells,^29^ we used an HMGB1-neutralizing antibody to neutralize potential HMGB1 in the culture supernatant. Notably, even after HMGB1 neutralization, Neo-2/15 also could maintain STAT5 phosphorylation in NK-92 (Supplementary Fig. 1g). Similarly, Neo-2/15 increased the expression of the proliferation marker Ki67 and effectors PFN and GrB in NK-92 compared to other agonists (Fig.1i and Supplementary Fig. 2a). Moreover, Neo-2/15 treatment exhibited lower levels of inhibitory receptors (Fig. 1j and Supplementary Fig. 2b), higher expression of the NKG2D and CD16 (Fig. 1k and Supplementary Fig. 2c) in NK cells, which corresponded to the second-highest cytotoxicity to K562 cells (Fig. 1l and Supplementary Fig. 2d, e). Taken together, Neo-2/15 effectively facilitated the proliferation, augmented the cytotoxic capabilities, and mitigated the exhaustion of NK cells, contributing to a favorable antitumor response. Consequently, we proceeded to test Neo-2/15 as a CAR-NK armor that prolonged the immune activation and resist the TME immunosuppression-induced functional impairment.

### Neo-2/15 potentiates the antitumor efficacy of CAR-NK cells

**Fig. 2:**
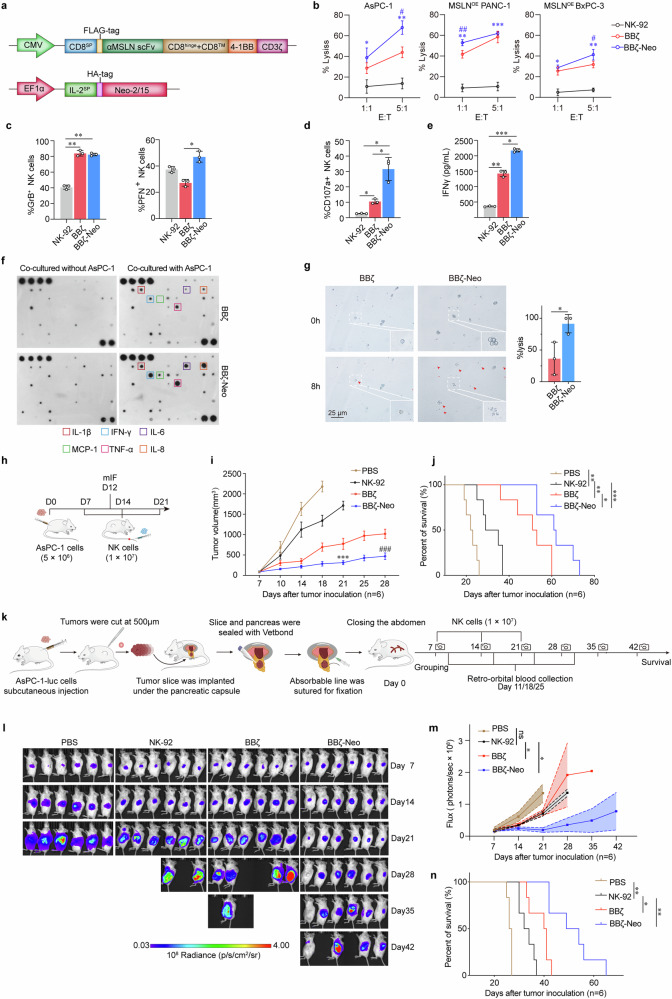
Neo-2/15-armored CAR-NK cells exhibit potent antitumor efficacy in vitro and in vivo. **a** Schematic representation of the lentivirus vectors encoding the αMSLN-CAR (upper) and Neo-2/15 (lower). Transmembrane region: CD8, co-stimulating domain: 4-1BB and CD3ζ, promoter: CMV or EF1α, SP: signal peptide. **b** Cytotoxicity of NK-92, BBζ and BBζ-Neo toward AsPC-1, MSLN^OE^ PANC1 or MSLN^OE^ BxPC-3 cells after co-culture for 4 hours at E:T ratios of 1:1 or 5:1 detected by the LDH assay. Data are presented as mean ± SD (*P < 0.05, **P < 0.01, ***P < 0.001 comparing BBζ-Neo with NK-92, ^#^*P* < 0.05, ^##^*P* < 0.01, comparing BBζ-Neo with BBζ, *n* = 4). Quantification of the expression of PFN and GrB (**c**) and CD107a (**d**) in NK-92, BBζ or BBζ-Neo after co-cultured with AsPC-1 cells for 4 hours. Data are presented as mean ± SD (*P < 0.05, **P < 0.01, *n* = 3). **e** IFNγ secreted by NK-92, BBζ or BBζ-Neo after co-cultured with AsPC-1 cells for 4 hours at the E:T ratio of 1:1 detected by ELISA. Data are presented as mean ± SD (*P < 0.05, **P < 0.01, ***P < 0.001, *n* = 3). **f** Cytokine array analysis of cytokines secreted by BBζ or BBζ-Neo after co-cultured with or without AsPC-1 cells for 4 hours. **g** Representative images of the morphology of PDO after 8-hour co-culture with BBζ, or BBζ-Neo at the E:T ratio of 4:1. Red arrows indicate PDO that are efficiently lysed. Insets show enlarged images (left). Lysis efficiency was assessed by enumerating the lysed cells within each field of view (right). Lysis (%) = (lysed cells / total cells) × 100%. Data are presented as mean ± SD (*P < 0.05, *n* = 3). **h** Schematic representation of the evaluation of antitumor activity of NK-92, BBζ or BBζ-Neo in vivo. NCG mice were engrafted with 5 × 10^6^ AsPC-1 cells on day 0. The tumor-bearing mice received NK-92, BBζ or BBζ-Neo (1 × 10^7^ per mouse) on day-7, -14 and -21. On the day-12 after tumor inoculation, some mice were euthanized and intratumoral NK cells were detected by multicolor immunofluorescence (mIF). Tumor growth (**i**) and survival (**j**) of tumor-bearing mice treated with PBS (control), NK-92, BBζ or BBζ-Neo. Data are presented as mean ± SD (ns, not significant, *P < 0.05, **P < 0.01, ***P < 0.001 compared with NK-92, ^###^*P* < 0.001 compared with BBζ, *n* = 6). **k** Schematic outline of the development of the modified orthotopic PDAC model and evaluation of the antitumor activity of various NK cells. The tumor-bearing mice received NK-92, BBζ or BBζ-Neo (1 × 10^7^ per mouse) on day-7, -14 and -21 (created using BioRender). **l** The tumor burden in tumor-bearing mice were monitored by measuring luminescence using IVIS. **m** Overall kinetics of systemic tumor progression in mice are plotted. Data are presented as mean ± SD (ns, not significant, *P < 0.05, *n* = 6). **n** Kaplan-Meier curve representing the percent survival of the indicated experimental groups. Statistics: two-tailed log rank test. Data are presented as mean ± SD (*P < 0.05, **P < 0.01, *n* = 6)

We generated MSLN-specific CAR-NK cells by transducing a lentiviral vector encoding anti-MSLN scFv fused with intracellular signaling domains of 4-1BB and CD3ζ into NK-92 cells (hereafter referred to as BBζ). BBζ were then transduced with another lentiviral vector encoding Neo-2/15 (hereafter referred to as BBζ-Neo) (Fig. 2a and Supplementary Fig. 3a, b). The cytotoxicity of BBζ and BBζ-Neo were measured by co-culturing them with MSLN-positive tumor cells (AsPC-1, MSLN^OE^ PANC-1 or MSLN^OE^ BxPC-3, Supplementary Fig. 3c) for 4 hours at an effector to target ratio (E:T) of 1:1 or 5:1. Compared to non-transfected NK-92 or BBζ, BBζ-Neo exhibited the strongest cytotoxicity (Fig. 2b). Moreover, following co-cultivation with AsPC-1 cells, GrB expression in BBζ-Neo or BBζ was higher compared to NK-92 (Fig. 2c and Supplementary Fig. 3d), and the expression of PFN or CD107a was increased in BBζ-Neo compared to BBζ or NK-92 (Fig. 2c, d and Supplementary Fig. 3d, e). Furthermore, BBζ-Neo expressed lower levels of immunosuppressive molecules compared to BBζ or NK-92 (Supplementary Fig. 3f). Additionally, when co-cultured with tumor cells, BBζ-Neo exhibited higher levels of IFNγ, IL-1β, IL-6, MCP-1, TNF-α and IL-8 compared to BBζ (Fig. 2e, f). In parallel to assays using cell lines, we also used patients-derived organoids (PDO) established from PDAC with verified MSLN expression (Supplementary Fig. 3g, h). The PDO exhibited significant morphological collapse, indicative of cell death, when exposed to BBζ-Neo compared to BBζ (Fig. 2g).

To assess the in vivo antitumor activity of BBζ-Neo, we subcutaneously implanted NCG mice with AsPC-1 human PDAC cells, then infused these tumor-bearing mice either with PBS, as the control, or with NK-92, BBζ or BBζ-Neo 7 days later (Fig. 2h). Compared to infusion with BBζ or NK-92, treatment with BBζ-Neo led to the lowest tumor burden (Fig. 2i) and the longest survival (Fig. 2j), with increased NK cell infiltration in tumors (Supplementary Fig. 4a, b). As expected, the infiltrating NK cells exhibited higher levels of the activation marker CD107a and lower expression of the exhaustion marker TIGIT (Supplementary Fig. 4a–d). To better mimic the PDAC TME,^30^ we further assessed the efficacy of these NK cells utilizing an orthotopic model (Fig. 2k). Consistently, BBζ-Neo treatment resulted in the lowest tumor growth and the longest survival of tumor-bearing mice among the control and other NK cell infusions (Fig. 2l–n). Additionally, BBζ-Neo also effectively impeded the tumor growth and improved the survival of mice bearing xenograft tumors derived from MSLN-positive, Caov-3 human ovarian cancer cells (Supplementary Fig. 4e–g). These findings indicated that Neo-2/15 enhanced the effectiveness of CAR-NK against MSLN-positive solid tumors in vivo.

### Neo-2/15 empowers CAR-NK cytotoxicity through STAT5/Akt/c-Myc pathway

**Fig. 3:**
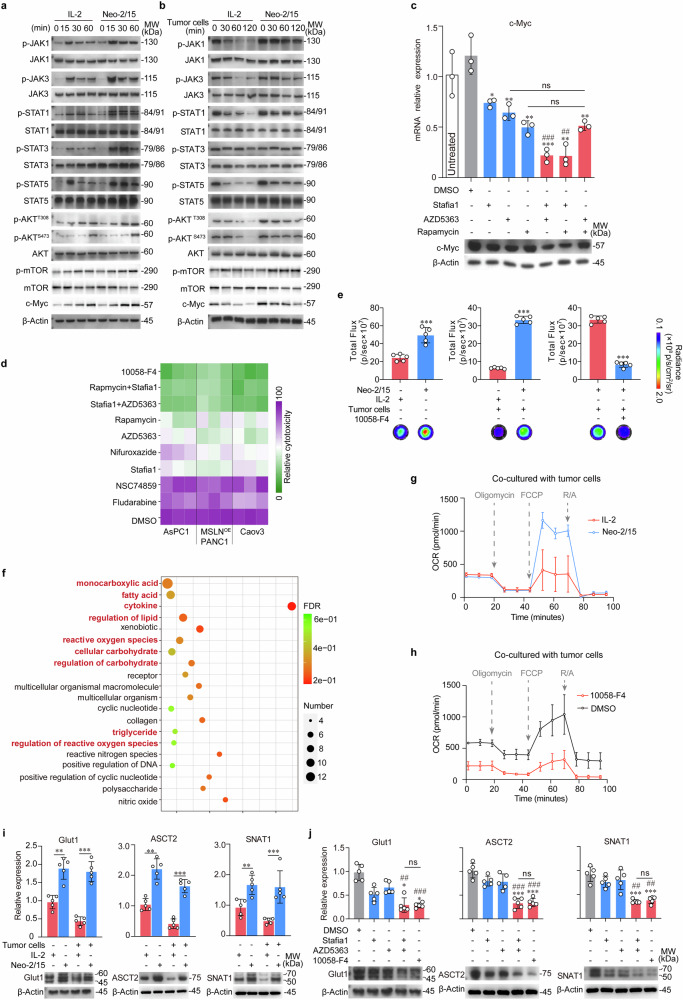
Neo-2/15 augments the expression of nutrient transporters and OXPHOS of CAR-NK cells. **a**, **b** Immunoblot for JAKs/STATs and Akt/mTOR pathways in BBζ stimulated with IL-2 or Neo-2/15 in the absence (**a**) or presence (**b**) of AsPC-1 cells. **c** The mRNA (top) and protein (bottom) levels of c-Myc in Neo-2/15 stimulated BBζ pretreated with Stafia1 (22 μM), AZD5363 (8 nM) or Rapamycin (10 nM) alone or in combination in the same concentration overnight were evaluated. Western blots are representative of three independent experiments. Data are presented as mean ± SD (ns, not significant, *P < 0.05, **P < 0.01, ***P < 0.001 compared with DMSO group, ^##^*P* < 0.01, ^###^*P* < 0.001 compared with Stafia1 group, *n* = 3). **d** Heatmap showing the cytolytic activity against MSLN positive tumor cells of Neo-2/15 stimulated BBζ pretreated with Fludarabine (50 μM), NSC74859 (86 μM), Stafia1 (22 μM), AZD5363 (8 nM), Rapamycin (10 nM), 10058-F4 (60 μM), Nifuroxazide (3 μM), Stafia1 (22 μM) + AZD5363 (8 nM), or Rapamycin (10 nM) + Stafia1 (22 μM) overnight measured by the LDH assay. **e** ATP generation in BBζ stimulated with IL-2 or Neo-2/15 without (left) or with (middle) co-cultured with AsPC-1 cells for 4 hours or in Neo-2/15 stimulated BBζ pretreated with 10058-F4 (60 μM) for 30 minutes and then co-cultured with AsPC-1 cells for 4 hours (right). Data are presented as mean ± SD (***P < 0.001, *n* = 5). **f** Representation of pathways altered in IL-2 or Neo-2/15 stimulated BBζ based on transcriptome data. Pathways of interest are highlighted. **g** OCR was assessed using a seahorse analyzer in BBζ stimulated with either Neo-2/15 or IL-2 after co-culturing with AsPC-1 cells. **h** OCR was assessed in Neo-2/15 expanded BBζ pre-treated with or without 10058-F4 (60 μM) and subsequently co-cultured with AsPC-1 cells. **i** mRNA (top) and protein (bottom) levels of the nutrient transporters in Neo-2/15 or IL-2 stimulated BBζ without or with co-cultured with AsPC-1 cells. Blots are representative of three independent experiments. Data are presented as mean ± SD (**P < 0.01, ***P < 0.001, *n* = 5). **j** mRNA (top) and protein (bottom) levels of the nutrient transporters in Neo-2/15 stimulated BBζ pretreated with Stafia1 (22 μM), AZD5363 (8 nM) or 10058-F4 (60 μM) for 30 minutes after co-cultured with AsPC-1 cells. Blots are representative of three independent experiments. Data are presented as mean ± SD (ns, not significant, *P < 0.05, ***P < 0.001 compared with DMSO group, ^##^*P* < 0.01, ^###^*P* < 0.001 compared with Stafia1 group, *n* = 5)

We went further to assay IL-2R downstream signaling in BBζ upon stimulation of Neo-2/15 or IL-2. Both Neo-2/15 and IL-2 effectively activated JAK1/3-STAT1/3/5 and Akt-mTOR signaling pathways, and moderately upregulated c-Myc expression in BBζ (Fig. 3a). However, when co-cultured with AsPC-1 cells through the transwell chamber (just mimics of TME), the rates of attenuation of c-Myc expression and IL-2R downstream signaling in Neo-2/15-stimulated BBζ were slower compared to IL-2-stimulated BBζ (Fig. 3b and Supplementary Fig. 5a). Further, dual inhibitions of STAT5 and Akt or STAT5 and mTOR in Neo-2/15-stimulated BBζ notably diminished the expression of c-Myc (Fig. 3c). We then subjected Neo-2/15-stimulated BBζ to the inhibitor targeting STAT1 (Fludarabine), STAT3 (NSC74859), STAT5 (Stafia1), STAT1/3/5 (Nifuroxazide), Akt (AZD5363), mTOR (Rapamycin) or c-Myc (10058-F4) prior to co-culture with three MSLN positive tumor cell lines, and the results showed that the cytolytic activity of Neo-2/15-stimulated BBζ was nearly abolished by c-Myc inhibitor or dual inhibitions of STAT5 and Akt or STAT5 and mTOR (Fig. 3d). Collectively, Neo-2/15 augments CAR-NK cell cytotoxicity by intensifying STAT5 and Akt activation, with c-Myc playing an essential role as the dominant downstream effector in this signaling cascade.

Then, we wanted to know how the sustained expression of c-Myc contributes to the cytotoxicity in Neo-2/15-stimulated BBζ within the TME. c-Myc, a key regulator of metabolism in lymphocytes and tumor cells, has been well-established.^31,32^ We subsequently analyzed the metabolic profile of BBζ stimulated with either IL-2 or Neo-2/15. Figure 3e showed that ATP production was significantly higher in the Neo-2/15-stimulated group compared to the IL-2-stimulated group, even when co-cultured with AsPC-1 cells. Notably, the inhibition of c-Myc markedly reduced the ATP level in Neo-2/15-stimulated BBζ which were co-cultured with AsPC-1 cells (Fig. 3e). These results suggested that c-Myc played a crucial role in Neo-2/15-mediated ATP generation by CAR-NK cells even in TME.

We then conducted the transcriptome analysis on BBζ stimulated with Neo-2/15 versus IL-2 upon co-culture with AsPC-1 cells. Gene ontology enrichment analysis (GO analysis) of differentially expressed genes revealed significant differences in metabolic pathways (Supplementary Fig. 5b), especially those for carbohydrate metabolism, regulation of carbohydrate, as well as cytokines and reactive oxygen species (ROS) (Fig. 3f). Furthermore, the non-targeted metabolomics analysis revealed the pantothenate and CoA biosynthesis, the AMPK signaling pathway, the pyruvate metabolism and the TCA cycle were the main pathways altered in Neo-2/15 expanded BBζ compared to IL-2-stimulated ones (Supplementary Fig. 5c). Additionally, there was a increase in the expression of enzymes associated with glycolysis and the TCA cycle, including HK2, ALDOA, PGK1, ENO1, PKM, ACLY and SUCLG1 (Supplementary Fig. 5d), and elevated levels of catabolic intermediates, such as fumarate and F6P, and the mitochondrial respiratory chain complex, especially complex I, in Neo-2/15 expanded BBζ (Supplementary Fig. 5e, f).

The TCA cycle is responsible for generating substantial quantities of NADH, which in turn facilitates the production of ATP through OXPHOS.^19^ Then, we assessed the level of OXPHOS by measuring the cellular oxygen consumption rate (OCR). When co-cultured with AsPC-1 cells, the mitochondrial maximal respiration of BBζ stimulated with IL-2 was compromised, whereas that of BBζ stimulated with Neo-2/15 was not (Fig. 3g and Supplementary Fig. 5g). Moreover, c-Myc inhibitor notably reduced OXPHOS, accompanied by significantly decreased basic respiration, in Neo-2/15-stimulated BBζ (Fig. 3h and Supplementary Fig. 5h). These data indicated the key role of c-Myc in maintaining the OXPHOS of Neo-2/15-stimulated BBζ for ATP production.

Glucose and glutamine are essential fuel sources for sustaining mitochondrial OXPHOS in activated NK cells.^20^ Then, we investigated the expression of the glucose and glutamine transporter. As shown in Fig. 3i and Supplementary Fig. 6a, the expression of Glut1, ASCT2 and SNAT1 was increased in Neo-2/15-stimulated BBζ, even when co-cultured with AsPC-1 cells compared to IL-2-stimulated BBζ. Consistently, inhibiting c-Myc or dual blockade of STAT5 and Akt profoundly diminished the upregulations of Glut1, ASCT2 and SNAT1 in Neo-2/15-stimulated BBζ which were co-cultured with AsPC-1 cells (Fig. 3j). The Glut1 expression changes in BBζ were also confirmed by flow cytometry (Supplementary Fig. 6b, c). Collectively, these results (summarized in Supplementary Fig. 6d) suggested that Neo-2/15 could enhance nutrient uptake and energy generation through STAT5/Akt/c-Myc pathway, potentially contributing to the potent cytotoxic effects of BBζ within the TME.

### Neo-2/15 maintains mitochondrial fitness of CAR-NK cells via c-Myc

**Fig. 4:**
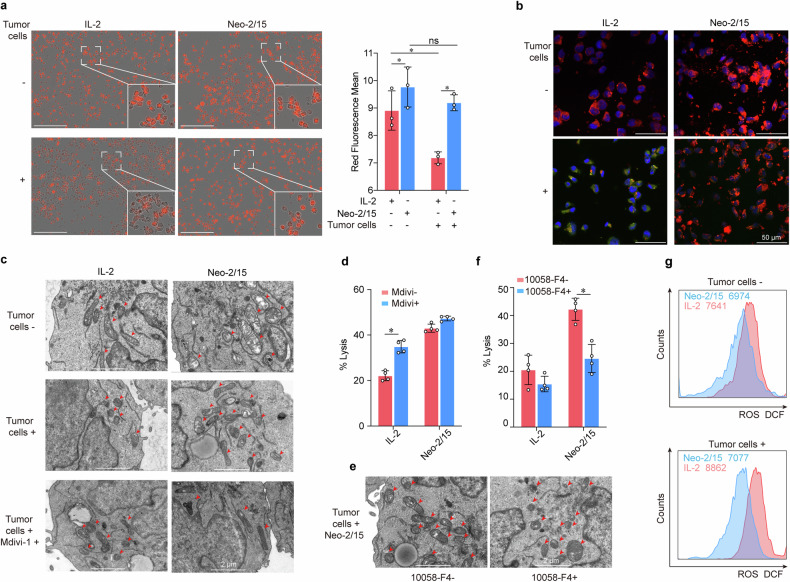
Neo-2/15 maintains mitochondrial fitness of CAR-NK cells within TME. **a**, **b** BBζ after being stimulated with IL-2 or Neo-2/15 and co-cultured with AsPC-1 cells or not at the E:T ratio of 1:1 for 4 hours is incubated with Mitotracker red (**a**) or JC-1 (**b**). For panel (**a**), data were analyzed using IncuCyte real-time imaging system to detect and quantify Mitotracker Red of mitochondrial image. Data are presented as mean ± SD (ns, not significant, *P < 0.05, *n* = 3). Scale bar = 200 μm. For panel (**b**), JC-1 aggregates are shown as red (indicating healthy mitochondria) whereas monomers are shown as green (indicating loss of mitochondrial membrane potential), and nuclei are stained with DAPI and are showing as blue. **c** Representative TEM images of BBζ treated with IL-2 or Neo-2/15 in the absence (top) or presence of AsPC-1 cells (middle), or with Mdivi-1 pretreatment and co-cultured with AsPC-1 cells (bottom). **d** IL-2 or Neo-2/15 stimulated BBζ pretreated with 10 nM Mdivi-1 and co-cultured with AsPC-1 cells for 4 hours at the E:T ratio of 1:1. The cytotoxicity is evaluated by the LDH assay. Data are presented as mean ± SD (*P < 0.05, *n* = 4). **e** Representative TEM images of BBζ treated with Neo-2/15 and co-cultured with AsPC-1 cells in the absence or presence of 10058-F4 pretreatment. Red arrows denote mitochondria. **f** IL-2 or Neo-2/15 stimulated BBζ pretreated with 60 μM 10058-F4 and co-cultured with AsPC-1 cells for 4 hours at the E:T ratio of 1:1. The cytotoxicity is evaluated by the LDH assay. Data are presented as mean ± SD (*P < 0.05, *n* = 4). **g** BBζ treated with IL-2 (red) or Neo-2/15 (blue) and co-cultured without (top) or with (bottom) AsPC-1 cells are stained with DCFH-DA and analyzed by flow cytometry

Then, we wondered how about the mitochondrial function of Neo-2/15-armored CAR-NK cells within TME. We first detected the mitochondrial mass by Mitotracker staining of NK cells under the presence of tumor cells, which mimic the TME, and found that the mitochondrial mass significantly decreased in BBζ treated with IL-2 rather than Neo-2/15, when co-cultured with tumor cells (Fig. 4a). Confocal laser scanning microscopy revealed comparable findings, showing substantial mitochondria in the cytoplasm of Neo-2/15-stimulated BBζ (Supplementary Fig. 7a). Next, the mitochondrial membrane depolarization was assessed using JC-1 staining, and we showed that Neo-2/15 rather than IL-2 could preserve the mitochondrial membrane potential of BBζ when co-cultured with tumor cells (Fig. 4b and Supplementary Fig. 7b).

Furthermore, we also observed the mitochondrial morphology by transmission electron microscope (TEM), and the results showed that Neo-2/15-stimulated BBζ remained with intact mitochondria in the cytoplasm, whereas IL-2-stimulated BBζ had small, fragmented and distinct mitochondria when co-cultured with tumor cells (Fig. 4c). Mdivi-1, a mitochondria fragmentation inhibitor, improved mitochondrial morphology and enhanced the tumor-killing ability of IL-2-stimulated BBζ (Fig. 4c, d). Additionally, pre-treatment with c-Myc inhibitor substantially increased the fragmentation of mitochondria and reduced the cytotoxic activity in Neo-2/15-stimulated BBζ (Fig. 4e, f). Accordingly, the level of mitochondrial ROS was moderately changed in Neo-2/15-stimulated BBζ, whereas markedly enhanced in IL-2-stimulated BBζ when co-cultured with tumor cells (Fig. 4g). Collectively, these results suggested that Neo-2/15 could maintain mitochondrial fitness of CAR-NK cells under the presence of tumor cells.

### Neo-2/15 promotes the persistence of CAR-NK cells within tumor by resisting ER-stress-induced apoptosis via c-Myc

**Fig. 5:**
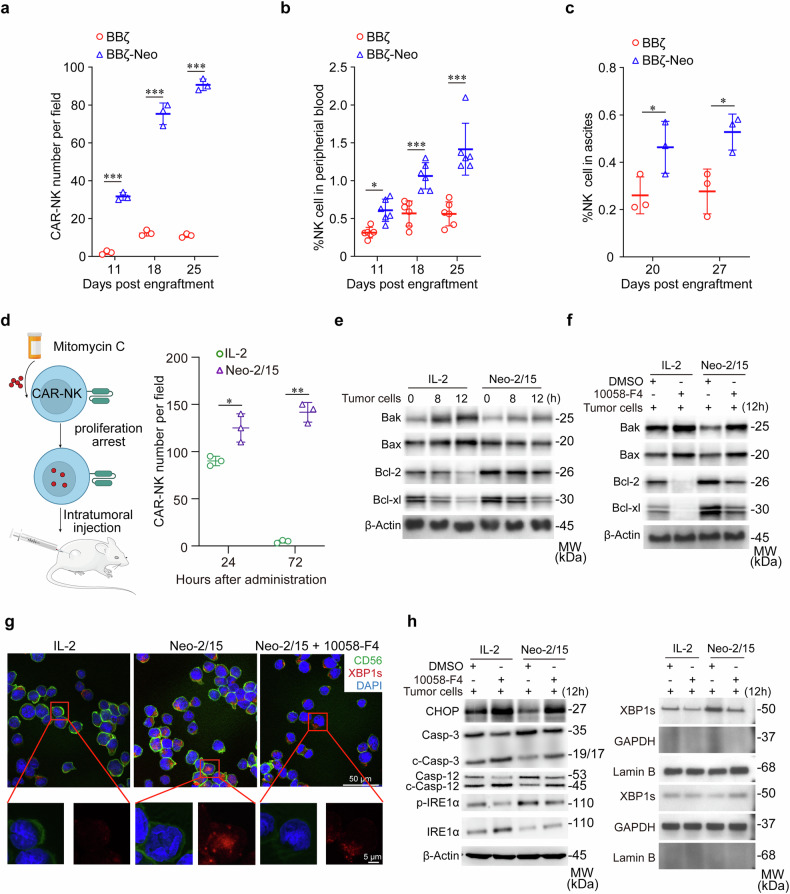
Neo-2/15 enhances the survival and persistence of CAR-NK cells in TME. **a** Quantification of CAR-NK cells in tumor tissues of orthotopic mice model. Data are presented as mean ± SD (***P < 0.001, *n* = 3). **b** Proportion of CAR-NK cells in the peripheral blood of mice in (**a**) was analyzed by flow cytometry. Data are presented as mean ± SD (*P < 0.05, ***P < 0.001, *n* = 6). **c** The number of CAR-NK cells in ascites was analyzed by flow cytometry. Data are presented as mean ± SD (*P < 0.05, *n* = 3). **d** Schematic of the intratumoral injection of CAR-NK cells treated with IL-2 or Neo-2/15 (left), and quantification of intratumoral BBζ at specified times post-administration (right). Data are presented as mean ± SD (*P < 0.05, **P < 0.01, *n* = 3). BBζ, stimulated with IL-2 or Neo-2/15 and pre-treated without (**e**) or with (**f**) 10058-F4, were co-cultured with tumor cells over specified durations. Western blot wase used to analyze the expression of Bax, Bak, Bcl-2, and Bcl-xl in BBζ. **g** Representation IF images for detecting the nuclear translocation of XBP1s in IL-2 or Neo-2/15 stimulated BBζ co-cultured with AsPC-1 cells for 8 hours at the E:T ratio of 1:1. XBP1s is marked in red, CD56 in green, and nuclei are stained with DAPI (blue). **h** IL-2 or Neo-2/15 expanded BBζ were pretreated with or without 10058-F4, following co-cultured with tumor cells for the 12 hours. Western blot analysis was performed to measure the expression of CHOP, caspase 3/12, total and phosphorylated IRE1α (left). XBP1s were analyzed in separated cytoplasmic and nuclear fractions, with GAPDH used as the cytoplasmic loading control and Lamin B used as the nuclear loading control (right)

As the TME-caused immunosuppressive polarization limits persistence of transferred CAR-NK cells in vivo, we next tested the kinetics of BBζ and BBζ-Neo in tumor tissues derived from tumor-bearing mice (Supplementary Fig. 8a). It was found that the number of BBζ-Neo was higher than that of BBζ in AsPC-1 xenograft lesions (Fig. 5a and Supplementary Fig. 8b), and the percentage of BBζ-Neo was higher than that of BBζ in peripheral blood (Fig. 5b). Moreover, the percentage of BBζ-Neo was also higher than those of BBζ in ascites of an ovarian cancer xenograft model (Fig. 5c and Supplementary Fig. 8c). Next, Neo-2/15- or IL-2-stimulated BBζ were treated with mitomycin-C to induce proliferation arrest, then were injected intratumorally into AsPC-1 xenografts (Fig. 5d left). Immunofluorescence (IF) analysis revealed that Neo-2/15 rather than IL-2 profoundly improved the persistence of BBζ in tumor tissues especially at 72 hours after intratumoral injection (Fig. 5d right and Supplementary Fig. 8d). Collectively, these data indicated that Neo-2/15 could promote the persistence of CAR-NK cells within the TME.

Mechanistically, compared to IL-2 treatment, Neo-2/15 stimulation significantly reduced the levels of the pro-apoptotic proteins Bax and Bak, and increased the levels of the anti-apoptotic molecules Bcl-2 and Bcl-xl in BBζ co-cultured with tumor cells (Fig. 5e). As anticipated, inhibiting c-Myc abrogated this effect (Fig. 5f), indicating that Neo-2/15 preserves the anti-apoptotic phenotype of BBζ in the TME via c-Myc. Moreover, Neo-2/15 markedly promoted spliced X-box binding protein 1 (XBP1s) translocation to the nucleus of BBζ when co-cultured with AsPC-1 cells (Fig. 5g). Also, Neo-2/15 downregulated CHOP expression and caspase-3/12 cleavage in BBζ, yet, these effects were blocked by the c-Myc inhibitor treatment (Fig. 5h). Besides, the c-Myc inhibitor abrogated the Neo-2/15-induced inositol-requiring enzyme 1α (IRE1α) phosphorylation and reduced the nuclear translocation and expression of XBP1s, which was consistent with the results of IF (Fig. 5g, h). Taken together, these findings suggested that Neo-2/15 could protect CAR-NK cells against endoplasmic reticulum (ER) stress-induced apoptosis via c-Myc.

### Neo-2/15 enhances CAR-NK ATP generation and antitumor efficacy via c-Myc/NRF1

**Fig. 6:**
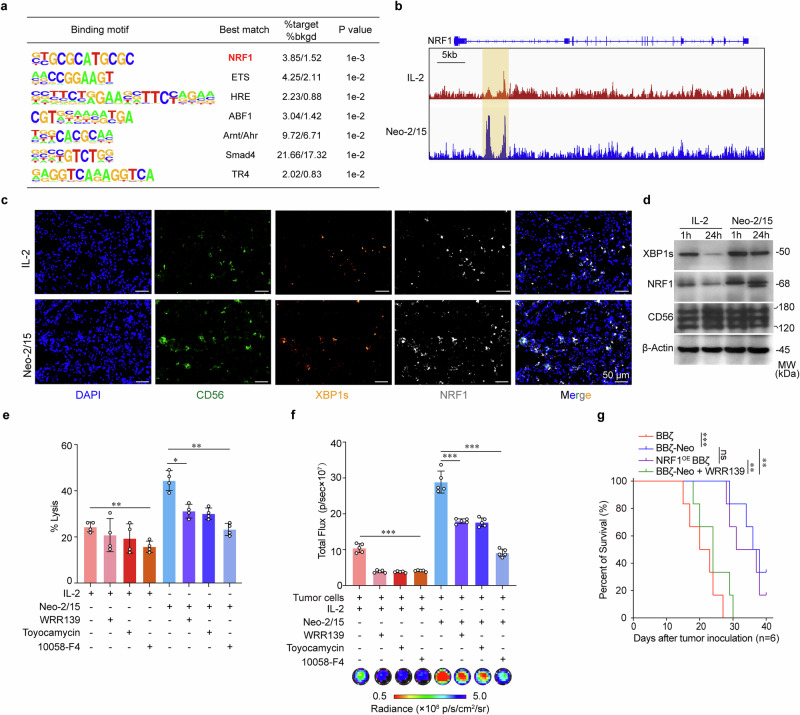
Neo-2/15 promotes ATP production and antitumor activity in CAR-NK cells via NRF1. **a** De novo motif analysis of XBP1s peaks at regions of open chromatin. “%target” is number of target sequences with motif over total target sequences; “%bkgd” (%background) is the number of background sequences with motif over total background sequences. p values calculated using binomial distribution in HOMER. **b** Genome browser images of XBP1s binding sites in regions of open chromatin in the vicinity of the NRF1 gene. **c** The expression of XBP1s and NRF1 of IL-2 or Neo-2/15 stimulated BBζ in tumor tissues detected by multicolor IF. CD56 (green), XBP1s (orange), NRF1 (white), or nucleus (DAPI; blue). **d** BBζ expanded with IL-2 or Neo-2/15 were collected from AsPC-1 grafts at specified time points post-injection. Expression of XBP1s and NRF1 were assessed using western blot. Killing potential (**e**) and ATP generation (**f**) of IL-2 or Neo-2/15 stimulated BBζ pretreated with 10058-F4 (60 μM), Toyocamycin (80 nM) or WRP139 (40 μM, NRF1 inhibitor) for 30 minutes and then co-cultured with AsPC-1 cells for 4 hours at E:T of 1:1. Data are presented as mean ± SD (*P < 0.05, **P < 0.01, ***P < 0.001, *n* = 4 in **e** and *n* = 5 in **f**). **g** Kaplan-Meier curve representing the percent survival of the experimental groups. Data are presented as mean ± SD (ns, not significant, **P < 0.01, ***P < 0.001, two-tailed log rank test, *n* = 6)

To further elucidate how the nuclear translocation of XBP1s improves Neo-2/15-stimulated BBζ function, we first performed CUT&Tag using an antibody to XBP1s. It was found that XBP1s bound to NRF1 at sites of open chromatin in Neo-2/15-stimulated BBζ when co-cultured with AsPC-1 cells (Fig. 6a, b). We also noted that Neo-2/15 treatment resulted in an upregulation of XBP1s and NRF1 expression in BBζ relative to those treated with IL-2, as evidenced by multicolor IF staining (Fig. 6c). Western blot and IF staining of BBζ isolated after 24 hours of infiltration into the xenograft further revealed that Neo-2/15 preserved the expression of XBP1s and NRF1 in BBζ within the TME, but IL-2 could not (Fig. 6d and Supplementary Fig. 9a).

To demonstrate the functional roles of NRF1 in BBζ, NRF1 was stably overexpressed in BBζ (NRF1^OE^ BBζ). We found that NRF1 overexpression enhanced the antitumor efficacy of IL-2-stimulated BBζ more than that of Neo-2/15-stimulated BBζ both in vitro (Supplementary Fig. 9b) and in vivo (Supplementary Fig. 9c), accompanied by enhanced ATP generation (Supplementary Fig. 9d). In contrast, NRF1 inhibitor WRR139, as well as XBP1 cleavage inhibitor (toyocamycin) and c-Myc inhibitor, weakened the cytotoxic activity (Fig. 6e) and ATP generation (Fig. 6f) of BBζ-Neo. NRF1 inhibitor further increased the tumor burden and decreased the survival of tumor-bearing mice treated with BBζ-Neo (Fig. 6g and Supplementary Fig. 9c). Collectively, these results suggested that Neo-2/15 could maintain ATP generation through c-Myc/NRF1, consequently enhancing the functionality of CAR-NK cells to improve their therapeutic efficacy in solid tumors (Supplementary Fig.10).

## Discussion

The infiltration, viability and cytotoxicity of CAR-NK cells within TME correlate significantly with the eradication of solid tumors.^14^ CAR-host cells have been genetically modified to secrete chemokines for achieving the accumulation of these cells into tumor tissues, resulting in regression of solid tumors.^15^ CAR-NK cells combined with a cytokine-expressing oncolytic virus has been demonstrated to induce robust antitumor efficacy in glioblastoma.^33^ However, preserving the functionality of CAR-host cells in TME remains a major challenge. It has been demonstrated that the compromised metabolic states contribute to the decline of tumor-fighting capabilities of CAR-NK cells during tumor progression.^34,35^ The restricted nutrition is primarily due to a poor supply caused by the presence of myeloid-derived suppressor cells (MDSC) and tumor cells,^36,37^ consequently diminishing the tumor-fighting capacity and persistence of CAR-NK cells. Furthermore, the depletion of essential amino acids in the TME can induce immune cell anergy, resulting in their inability to exert cytotoxic effects.^38^ Hence, establishing CAR-host cells that can maintain their metabolic fitness while mediating effector functions is crucial for achieving effective therapeutic efficacy against solid tumors. In the present study, we demonstrate that CAR-NK cells fortified with Neo-2/15 have improved nutrient competitiveness, enhanced energy generation, increased survival and persistence within TME and peripheral circulation. Consequently, these findings provide substantial evidence of the metabolically modified efficacy in the adoptive cell therapy against solid tumor.

Cytokine-armored CAR-host cells have been demonstrated to improve the therapeutic efficacy against solid tumors by enhancing resistance to immunosuppressive polarization, and thereby promoting antitumor immune responses.^39–42^ Additionally, cytokines, such as IL-2, IL-15, IL-12, IL-21 and IL-18, have been recognized for their ability to enhance the proliferation and activation of NK cells and have been employed to expand primary NK cells or augment the cytotoxicity of memory-like NK cells against hematologic malignancies.^43–45^ However, the proliferation of NK cells in vitro induced by these natural cytokines is typically constrained. For instance, IL-15, a commonly used cytokine for supporting NK cells activation, has been shown to result in functional exhaustion of human NK cells.^24^ Neo-2/15 has been shown to induce potent immunotherapeutic effects with reduced toxicity when compared with natural IL-2R agonist.^46^ It also exhibits a higher affinity for IL-2Rβγ compared to natural cytokines and activates downstream signaling independent of IL-2Rα and IL-15Rα.^27^ We previously reported that feeder cells engineered to secrete Neo-2/15 exhibit significant potential in enhancing NK cell proliferation and function.^47^ Also, tissue immune microenvironment or niche signals are important factors in determining and mediating anti-tumor immune response.^48,49^ Further identification and spatiotemporal characteristics of cellular interactions in regional immunity, tissue microenvironmental signals and niche components will be helpful to the new therapeutic strategies against tumor and infection and to enhance clinical benefit. Our results revealed that Neo-2/15 could maintain NK cell STAT5/Akt signaling and hold the downstream c-Myc expression in TME. Neo-2/15 expanded CAR-NK cells increase the nutrient transporters, mitochondrial OXPHOS rate, and ATP generation, resulting in resistance to TME immunosuppression-induced functional impairment, and consequently exhibiting enhanced cytotoxicity, less exhaustion and longer persistence within the TME, thereby improving CAR-NK therapeutic efficacy in pancreatic cancer and ovarian cancer.

Sustained mitochondrial mass is fundamental to OXPHOS, maximal respiratory capacity and mitochondria fusion increases the energy production in response to stress regardless of the cell type.^24^ The mitochondrial quality regulates CD8^+^ T cells fate decision, antitumor immunity, memory formation and metabolic fitness.^50^ Exhausted NK cells isolated from tumor-infiltrating lymphocytes of liver cancer mainly have fragmented and distinct mitochondria, accompanied with reduced cytotoxicity.^51^ OXPHOS in lymphocytes increased mitochondrial fitness, resulting in apoptotic resistance and cellular persistence in TME.^52^ Further, the fragmented mitochondria lead to cell dysfunction and silencing genes related to mitochondrial fusion could irreversibly block the immortalization of cancer stem cells and inhibit tumor formation.^53^ Hence, mitochondrial fitness and energy production are coupled with fates of immune and cancer cells. There are not many studies on the relationship between NK cell mitochondrial status, dynamic regulation and NK cell-mediated immune surveillance. At present, methods such as using mitochondrial fragmentation inhibitors like Mdivi-1 or knocking out mitochondrial fission genes are used to restore the mitochondrial morphology and the killing function of NK cells.^51^ Our results demonstrate that Neo-2/15 can maintain the mitochondrial fitness within TME, contributing to increased energy generation and anti-apoptotic capacity. We verified that the upholding of the mitochondrial fitness by Neo-2/15 is partially dependent on c-Myc upregulation. When c-Myc is chemically inhibited, Neo-2/15-armored CAR-NK cells exhibit mitochondrial fission, diminished OXPHOS, and reduced ATP generation, resulting in decreased anti-apoptotic phenotype and unsuccessful antitumor effects. These results provide evidence for optimizing the metabolism of engineered NK cells can invigorate their antitumor efficacy once transfused into tumor-bearing host.

c-Myc is demonstrated to be important for the gene expression and biogenesis of mitochondria.^54^ Additionally, the IRE1α-XBP1 pathway drives NK cell responses against tumors in vivo.^55^ Here we find that IRE1α and XBP1s are up-regulated and that XBP1s is translocated into the nucleus in Neo-2/15-stimulated CAR-NK cells, both of which are reduced when c-Myc is inhibited. We further confirm that NRF1 is the major transcriptional target involved in the regulation of XBP1s-mediated gene expression in Neo-2/15-treated CAR-NK cells, enhancing cytotoxicity both in vivo and in vitro. NRF1 influences cellular metabolism by inducing the transcription of diverse genes associated with mitochondrial functions, particularly those crucial for cellular respiration, ATP synthesis, and antioxidant defense across various cell types like hepatocytes.^56^ Our results also demonstrate that NRF1 overexpression enhanced the antitumor efficacy and ATP generation of IL-2-stimulated BBζ. NRF1 as well as c-Myc inhibitor, weakened the cytotoxic activity of BBζ-Neo. The inhibition of NRF1 leads to a significant decrease in ATP production in CAR-NK cells and survival of tumor-bearing mice treated with BBζ-Neo. Hence, we posit that effector cells with adequate energy can evade apoptosis within the TME and optimally perform their cytotoxic functions.

Collectively, the results of these studies highlight the optimization of therapeutic NK cell metabolism by Neo-2/15 for improved function within the metabolically adverse TME may provide a potential general strategy for metabolically modifying NK cell activity against immunosuppressive polarization in solid tumors.

## Methods

### Human samples

All human tumor tissues and peripheral blood used in this study were obtained under the approval of the Scientific Investigation Board of Navy Medical University (Approval number: CHEC2018-111, CHEC2024-109). PBMC samples from organ transplant recipients were collected from affiliated hospital of Navy Medical University, as reported previously.^57^ All other tumor tissue samples and PBMC samples were obtained were collected from the First Affiliated Hospital of Navy Medical University (Shanghai, China). Patients were included based on the primary disease and no drug treatment before resection.

### Mice and cell lines

Female NOD/ShiltJGpt-*Prkdc*^*em26Cd52*^*Il-2rg*^*em26Cd22*^/Gpt (NCG) mice were obtained from Gempharmatech Inc (Nanjing, China) and housed under SPF facilities. Mice experiments were approved by the Scientific Investigation Board of Navy Medical University (Shanghai, China). NK-92 cell line was cultured as previously described.^7^ K562 chronic myeloid leukemia cell line was obtained from the Immocell company (Immocell Biotechnology Co., Ltd., Xiamen, China). Caov-3 ovarian cancer cell line was obtained from the Shanghai Cell Bank (Chinese Academy of Sciences, Shanghai, China). The human pancreatic cancer cell lines (AsPC-1, PANC-1 and BxPC-3) were gifts from Department of Pathology, Changhai Hospital, Navy Medical University. MSLN overexpressing cell lines (MSLN^OE^ PANC-1 and MSLN^OE^ BxPC-3) were generated by lentiviral transduction (Genepharma, LV2021-11386). Caov-3 and AsPC-1 were labeled with luciferase (Genechem, GV633) as previously described.^47^ The pancreatic cells were cultured in DMEM medium (Gibco), containing 10% fetal bovine serum (FBS). All cell lines were tested negative for mycoplasma.

### NK cell proliferation and treatment

IL-2 and IL-15 were from NovoProtein. The superkines used in NK expansion, including Neo-2/15, H9, H9T, and N72D, were expressed and purified by NovoProtein and underwent endotoxin quality control using the Limulus Amebocyte Lysate (LAL) assay to ensure they met acceptable endotoxin levels. Primary NK cells were isolated and cultured in AIM-V medium as we previously described.^47^ Specifically, primary NK cells were stimulated with different superkine agonists (final concentration of 1 nM) for 21 days. IL-2, H9, H9T NK-92 cells were incubated with increasing concentrations (0-2 nM) of different superkine for 7 days at 37 °C. The EC_50_ was determined with the dose-response curve generated from the experimental data by nonlinear regression variable slope curve-fitting with GraphPad Prism8. For inhibition of NK cells, vehicle control (DMSO), Mdivi-1 (10 nM) was added to cell supernatant of NK cells culture media.

### Western blot

Cells were lysed with ice-cold protease inhibitors in RIPA lysis buffer. Total protein concentration was quantified with bicinchoninic acid (BCA) assay, size fractionated by polyacrylamide gel electrophoresis, and transferred to nitrocellulose membranes. After blocking with TBST solution containing 5% BSA, membranes were probed with indicated primary and secondary antibodies. Blots were visualized using Tanon imaging system (4600SF). Loaded samples were normalized by β-Actin staining.

### ELISA

Specific steps as we previously described.^58^ In brief, 4 hours co-culture of CAR-NK cells with AsPC-1 or organoids, the supernatant was collected and analyzed using the quantizing ELISA human IFNγ Immunoassay according to the manufacturer’s instructions (R&D, DIF50C). A microplate reader (PerkinElmer, Waltham, MA, USA) was used to read the 96-well plates at 450 nm.

### Flow cytometry

Specific steps as we previously described.^59^ For cell surface staining, the single-cell suspensions were incubated with CD107a-FITC or FLAG tag-PerCP/Cyanine5.5 antibody for 20 minutes at 4 °C. At the completion of incubation, cells were washed with FACS buffer. For intracellular staining, cells were fixed with fixation buffer for 5 minutes on ice, followed by permeabilization with perm/wash buffer overnight at 4 °C, then finally washed before analysis. For JC-1 assays to assess mitochondrial membrane potential, cells were lifted from culture plates and stained for 30 minutes at 37 °C in 1 mM JC-1 dye, washed and analyzed on an FACSDiva software (BD Biosciences). Aggregates were measured under Texas Red and monomers under FITC.

### Construction and culture of PDO

The organoids from pancreatic cancer patients were established and maintained as described previously.^60^ In brief, tumor tissue was minced and digested with collagenase II (5 mg/mL) at 37 °C for 1 hour. Then embedded in matrigel BME2, and cultured in human complete medium [AdDMEM/F12 medium supplemented with HEPES (1×), Glutamax (1×), penicillin/streptomycin (1×), B27 (1×), Primocin (1 mg/mL), N-acetyl-L-cysteine (1 mM), Wnt3a (30 ng/mL), Y27632 (10 μM), Forskolin (10 μM), RSPO1 (250 ng/mL), Noggin recombinant protein (25 ng/mL), epidermal growth factor (EGF, 50 ng/mL), Gastrin (10 nM), FGF10 (100 ng/mL), Nicotinamide (10 mM) and A83-01 (0.5 μM)]. Images of growing organoids were obtained using the DP73 microscope digital camera at the same positions.

### Cytotoxicity assay

The cytotoxicity function of CAR-NK cells was assessed by co-culture CFSE-labeled tumor cells at an E:T ratio of 1:1. CAR-NK cells and target cells were co-cultured for 4 hours. Following co-culture, cells were stained with 7-AAD to identify dead cells. Flow cytometric analysis was performed to distinguish cell populations: CFSE^+^7-AAD^-^ cells were defined as non-killed target cells, whereas CFSE^+^7-AAD^+^ cells represented target cells that had been killed. To ensure accurate enumeration, a set time-volume acquisition strategy was employed. Specifically, Lysis (%)=[Initial Total Cells−CSFE^+^7-AAD^-^ Cells]/ Initial Total Cells × 100%. For lactate dehydrogenase (LDH), CytoTox96 cytotoxicity assay was used according to the manufacturer’s instructions (Promega, G1780). Lysis (%) = [LDH^E:T^ -LDH^E^]/LDH^Max^× 100%. Specific steps as we previously described.^30^

For organoid cytotoxicity assays, specific steps as previously described.^61^ Briefly, organoids were seeded and incubated with CAR-NK cells as follows: culture plates were first moistened using culture medium and then covered with 35 μL matrigel. Confluent organoids were collected and added to matrigel covered wells. Organoids were grown for 24 hours before addition of CAR-NK cells in 500 μL of medium. The images were acquired at identical positions using the microscope digital camera.

### Generation of CAR-NK cells

Autocrine Neo-2/15-expressing CAR-NK cells were generated through two rounds of lentiviral transduction. In the procedure, we utilized two distinct lentiviral preparations: one encoding the CAR construct and the other encoding Neo-2/15. The MSLN-specific CAR and Neo-2/15 sequences were cloned into the pLVX-EF1a-IRESPuro vector (Addgene, 85132). Lentivirus was produced by co-transfecting lenti-X-293 cells with the packaging plasmids pCMVR8.74 and pMD2.G. For the first transduction, NK-92 cells were infected with the CAR-encoding lentivirus at an MOI of 10, in the presence of 10 μg/mL polybrene to enhance transduction efficiency. The cells were incubated with the lentiviral particles for 72 hours. After obtaining monoclonal CAR-expressing NK-92 cells, they underwent a second transduction using the Neo-2/15-encoding lentivirus at an MOI of 25. Transduced cells were sorted for Neo-2/15-positive populations using an HA tag, and single-cell clones were isolated via limiting dilution. To further refine the selection, individual clones were permeabilized following treatment with 25 mg/mL Brefeldin A, and the highest Neo-2/15-expressing clone was screened for following applications. The amino acid sequence of anti-MSLN CAR and Neo-2/15 are provided in Supplementary Table 1.

### Cytokine membrane array

Specific steps were previously described.^30^ Briefly, collected supernatant of BBζ and BBζ-Neo after incubation with AsPC-1 cells for 4 hours, followed by 10,000 G centrifugation for 10 minutes. The protocol was performed and analyzed according to the manufacturer’s instructions. Cytokines were individually mapped and both background and positive control values were marked.

### IF

Specific steps as previously described.^62^ For multicolor IF, tissues were formalin-fixed and paraffin-embedded. The staining was performed with a AlphaTSA automation multiplex Kit. Antigen retrieval was performed by using bond ER Solution followed blocking agents. Incubated with primary antibodies 30 minutes at 37 °C, followed with secondary antibodies at 37 °C for 10 minutes. To fluorescently label proteins, XTSA dye working solution were used at RT for 5 minutes. Counterstaining, mounting and imaging. Fluorescence images were scanned using a SP8 fluorescence confocal microscope (Leica) for cell sample or PathScan (Excilone) for tissue sample. These images were spectrally unmixed using single stain positive control images in the InForm software (Akoya Biosciences, Marlborough, Massachusetts, USA), and immune cells were quantified using HALO software (Indica Labs, Albuquerque, New Mexico, USA).

### Tumor-bearing mouse models and antitumor therapy

For the AsPC-1 subcutaneous tumor model, AsPC-1 cells (5 × 10^6^) were inoculated subcutaneously in the right flank of NCG mice. Tumors were established in mice and then treated with 1 × 10^7^ NK-92, BBζ and BBζ-Neo via tail vein injection on day 7, 14 and 21 after inoculation. Tumor volume was calculated by multiplying the tumor length and width three times a week, and mice reaching maximum tumor burden or exhibiting moribund signs were euthanized. Orbital bleed was collected to detect NK cell persistence by flow cytometry.

For the Caov-3 ovarian cancer xenograft model, 6-week-old female NCG mice were injected with 1 × 10^7^ luciferase-labeled Caov-3 cells into the peritoneal cavity. Then treated with 1 × 10^7^ NK-92, BBζ and BBζ-Neo via tail vein injection on day 7, 14 and 21 after inoculation. The growth of metastatic colonies was measured by luciferase-based bioluminescence imaging by using the platform of IVIS Lumina II (PerkinElmer). Tumor ascites was collected to detect NK cells persistence by flow cytometry.

Modified orthotopic PDAC model was established as previously reported.^30^ Briefly, luciferase-labeled AsPC-1 cells were inoculated subcutaneously in 6-week-old NCG mice (1 × 10^7^ cells in each mouse). Tumors were allowed to grow for 2-3 weeks until they were approximately 8 ~ 10 mm in diameter. Collected and sectioned tumor samples. Then, ophthalmic scissors were used to cut a 1 cm port above the spleen. The capsule of the pancreas was opened and lifted for inserting tumor slice into the subcapsular space. The graft was fitted to the pancreas and pancreas were incorporated into the abdominal cavity and the abdomen was closed. Different groups were visualized by IVIS Lumina II (PerkinElmer). Then treated with 1 × 10^7^ NK-92, BBζ and BBζ-Neo via tail vein injection on day 7, 14 and 21 after inoculation.

### RNA sequencing

Total RNA isolation, RNA quality control, library construction, sequencing and data analysis were performed by Shanghai Biotechnology Corporation (Shanghai, China). The differentially expressed genes were identified using edgeR. The *p*-value significance threshold in multiple tests was set by the false discovery rate (FDR). The set of potential target genes was screened using the following filter criteria: *q*-value < 0.05 and fold-change > 2. To identify differentially expressed genes implicated in specific biological processes, we employed the GO framework for annotation and classification.

### Metabolism assays

For seahorse metabolic assays, after co-cultured AsPC-1 cells with BBζ or BBζ-Neo were analyzed with an XF24 Extracellular Flux Analyzer (Seahorse Biosicence) as previously described.^58^ Briefly, mitochondrial stress test was performed using the sequential injection strategy, 1.5 μM oligomycin, 0.5 μM FCCP and 0.5 μM Rot/AA. Intracellular ATP concentrations were determined with an ATP Determination Kit according to the manufacturer’s instructions (Promega, G7573). Mitochondria membrane potential was determined using JC-1 assay kit (Invitrogen, T3168). Cellular mitochondria were stained with MitoTracker Red (Yeasen, 40741ES50) and determined with flow cytometry or IF and quantified by using incuCyte Zoom Live-content imaging system (SARTORIUS). For unbiased metabolomics analysis, BBζ (1 × 10^7^) were collected and subjected to metabolite profiling using liquid chromatography-mass spectrometry (LC/MS) system by Oebiotech, Shanghai, China. Data are analyzed using a Progenesis QI V2.3 system (Nonlinear, Dynamics, Newcastle, UK). For TEM, cell samples sent to Servicebio (Wuhan, China) for sample processing and electron microscope photography.

### CUT&Tag

The CUT&Tag assay was conducted using Hyperactive Universal CUT&Tag Assay Kit for Illumina. Briefly, BBζ (1 × 10^6^) were collected after co-culture with AsPC-1 cells and then bound to ConA beads for 10 minutes at 25 °C. Cells were incubated with XBP1s antibody at 4 °C overnight. Anti-mouse IgG was added and incubated for 1 hour at 25 °C followed incubation with 0.04 μM pA/G-Tnp for 1 hour at 25 °C and wash. Resuspended in tag mentation buffer and incubated at 37 °C for 1 hour. Tag mentation was stopped by adding proteinase K, buffer LB and DNA extract beads and incubated at 55 °C for 10 minutes, cells were plated on a magnet and unbound liquid was removed. Libraries were constructed using TD903 Hyperactive Universal CUT&Tag Assay Kit for Illumina, and TD202 TruePrep Index Kit V2 for Illumina (Vazyme Biotech). CUT&Tag data processing were performed by Shanghai Biotechnology Corporation. Macs (2.2.7.1) was used for peak calling. The set of potential peaks was screened using the following filter criteria: *p*-value < 0.05 and fold-change > 2.

### Statistical analysis

GraphPad Prism 8.0 was used for all statistical analyses. A two-tailed unpaired student t-test was used to determine significance. One-way analysis of variance (ANOVA) with a Bonferroni post-test was used to compare differences among multiple groups. Survival analysis was performed by Kaplan-Meier survival analysis. Schematic illustrations were created using BioRender (https://www.biorender.com/) and Adobe Illustrator (version 25.0; Adobe Inc.).

## Supplementary information

Supplemental material

## Data Availability

All reagents used in this study are either commercially available or can be made available from the corresponding author upon reasonable request. The RNA-seq data have been deposited in Gene Expression Omnibus (GEO) with GEO accession GSE243530. The CUT&Tag assay have been deposited in GEO with GEO accession GSE243385.
