# Supplementary material for: Neoleukin-2/15-armored CAR-NK cells sustain superior therapeutic efficacy in solid tumors via c-Myc/NRF1 activation
Source: Signal Transduct Target Ther. 2025 Mar 3;10:78. doi: 10.1038/s41392-025-02158-2 (PMC11873268; doi:10.1038/s41392-025-02158-2)
Supplement: Supplementary file 1 — Supplemental material [file 41392_2025_2158_MOESM1_ESM.doc]

**Supplementary Materials for**

**Neoleukin-2/15-armored CAR-NK cells sustain superior therapeutic efficacy in solid tumors via c-Myc/NRF1 activation**

Jianhua Luo^1,#^, Meng Guo^1,#^,*, Mingyan Huang^1,#^, Yanfang Liu^1,2^, Yuping Qian^2^, Qiuyan Liu^1^,* and Xuetao Cao^1,3,4^,*

^*^Correspondence to: Meng Guo ([guom@immunol.org](mailto:guom@immunol.org)), Qiuyan Liu ([liuqy@immunol.org](mailto:liuqy@immunol.org)), Xuetao Cao (caoxt@immunol.org)

**This file includes:**

Supplementary Figures 1-10 and Table 1


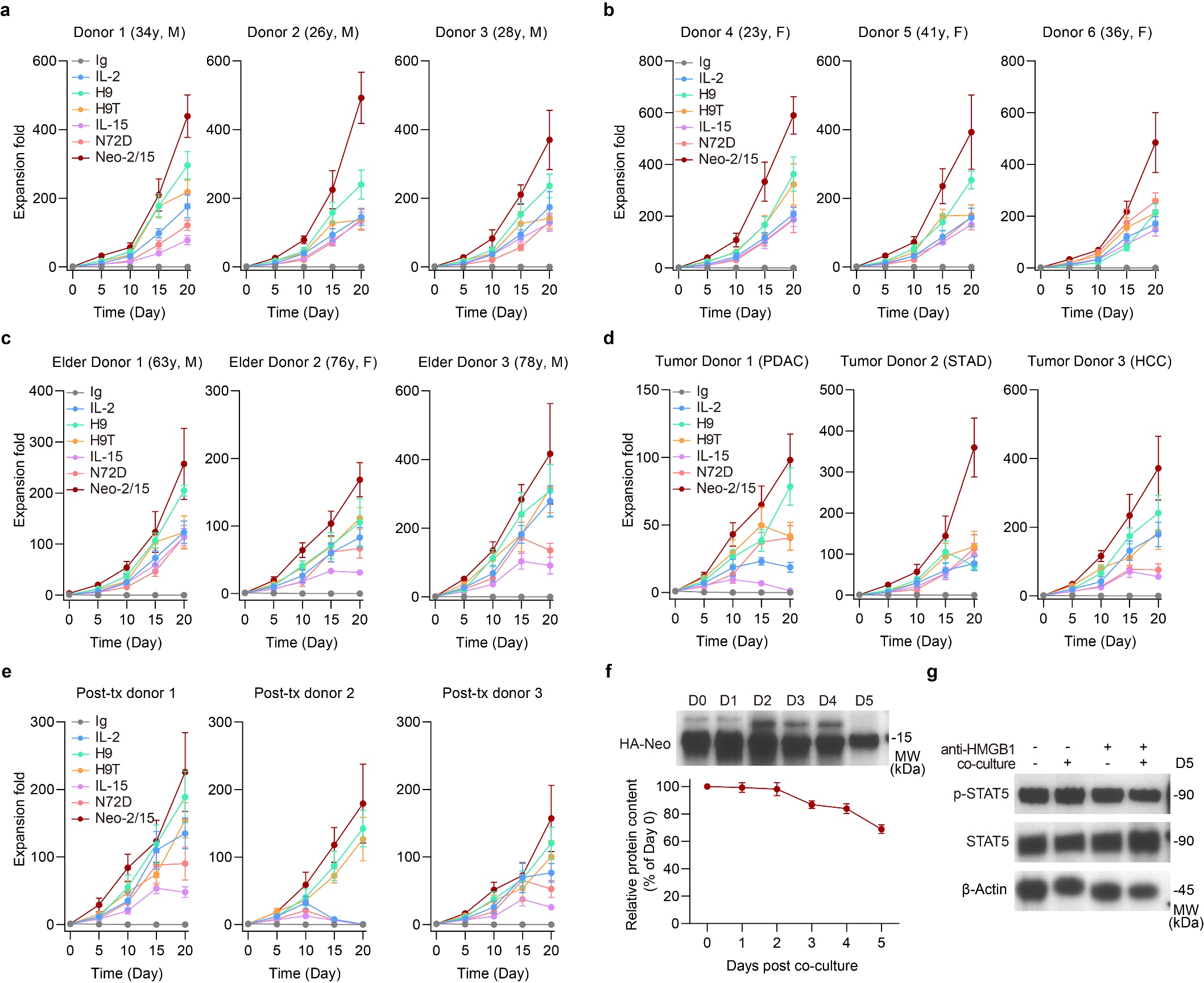


**Supplementary Fig.1** | **Neo-2/15 modulates the expansion and activation of NK cells.** **a-e**, Expansion analysis of primary NK cells from male donors (**a**), female donors (**b**), elderly donors (**c**), donors with malignancy (**d**), and organ transplant recipients being treated with immunosuppressants (**e**) stimulated with 1 nM indicated cytokine or superkine (a-e, n=6). PDAC: pancreatic ductal adenocarcinoma; STAD: stomach adenocarcinoma; HCC: hepatocellular carcinoma. **f**, 1 nM Neo-2/15 is added to the medium of AsPC-1 cell culture, the culture supernatant is collected 5 days later. The Neo-2/15 is detected by immunoblotting (top). The grayscale value of the western blot bands is shown (bottom). **g**, Neo-2/15 is added to the medium of AsPC-1 cell cultures, the culture supernatant is collected 5 days later. The supernatant was incubated with 10 µg/mL neutralizing anti-HMGB1 antibody for 1 hour, and then added to cytokine-free NK-92 culture medium at a 1:10 ratio. NK-92 cells are harvested 1 hour later, then the total and the phosphorylated STAT5 in cell lysates are detected by immunoblotting.


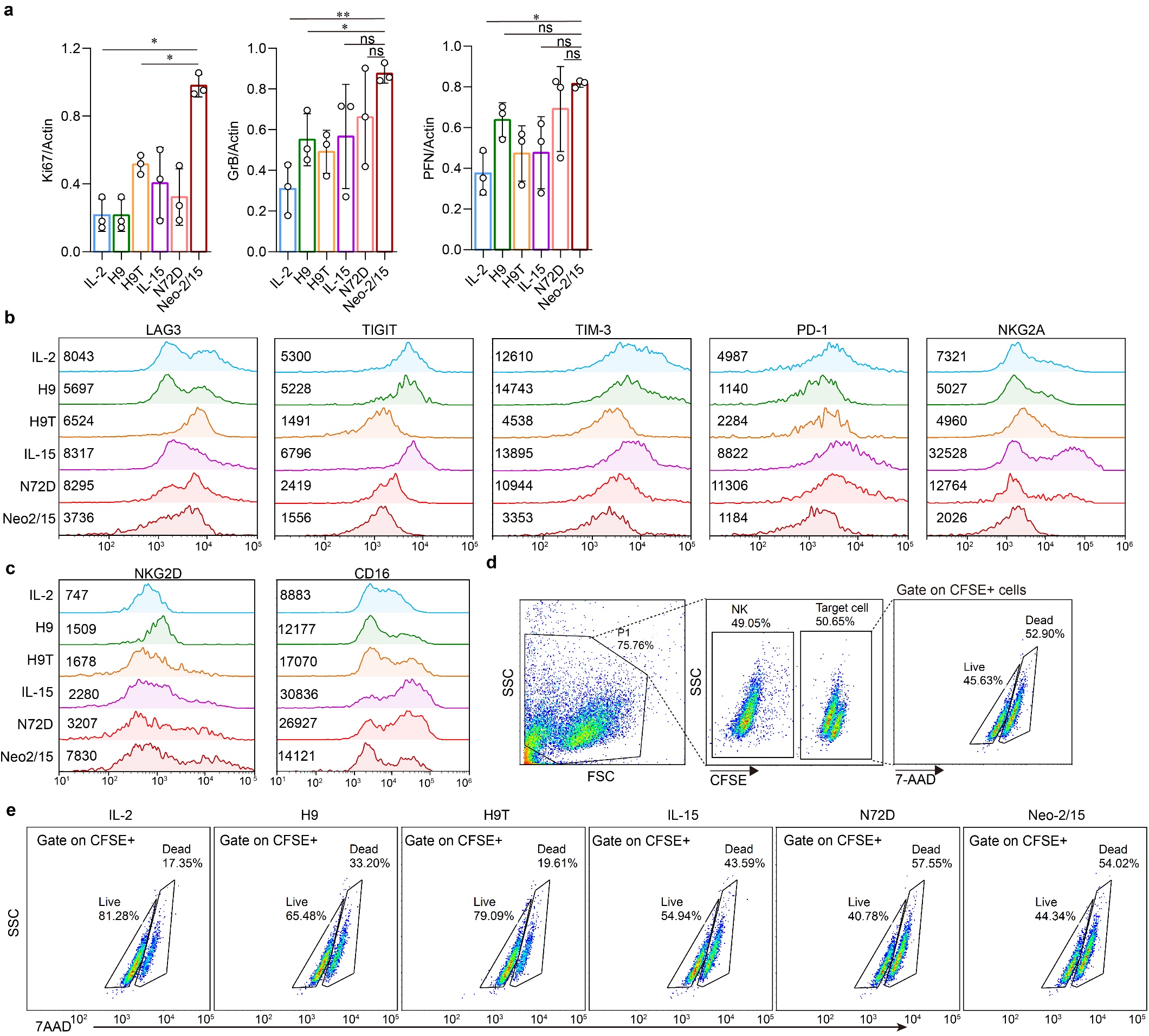


**Supplementary Fig.2** | **Neo-2/15 modulates the exhaustion and cytotoxicity of NK cells.** **a**, The grayscale value of the western blot bands shown in **Fig. 1i** is quantitatively represented across three independent experiments. Data are presented as mean ± SD. (ns, not significant, **P* <0.05, ***P*<0.01, n=3). **b-c**, Primary NK cells were stimulated with 1 nM indicated cytokine or superkine for 1 hour, the expression of LAG3, TIGIT, TIM-3, PD-1, NKG2A (**b**), NKG2D and CD16 (**c**) was analyzed by flow cytometry. **d**, Overview of gating strategy and cytotoxic function analysis. **e**, Representative fluorescence-activated cell sorting dot plot showing the killing activity of primary NK cells targeting K562 at an E:T ratio of 1:1. The dead target cells appear in the right quadrant.


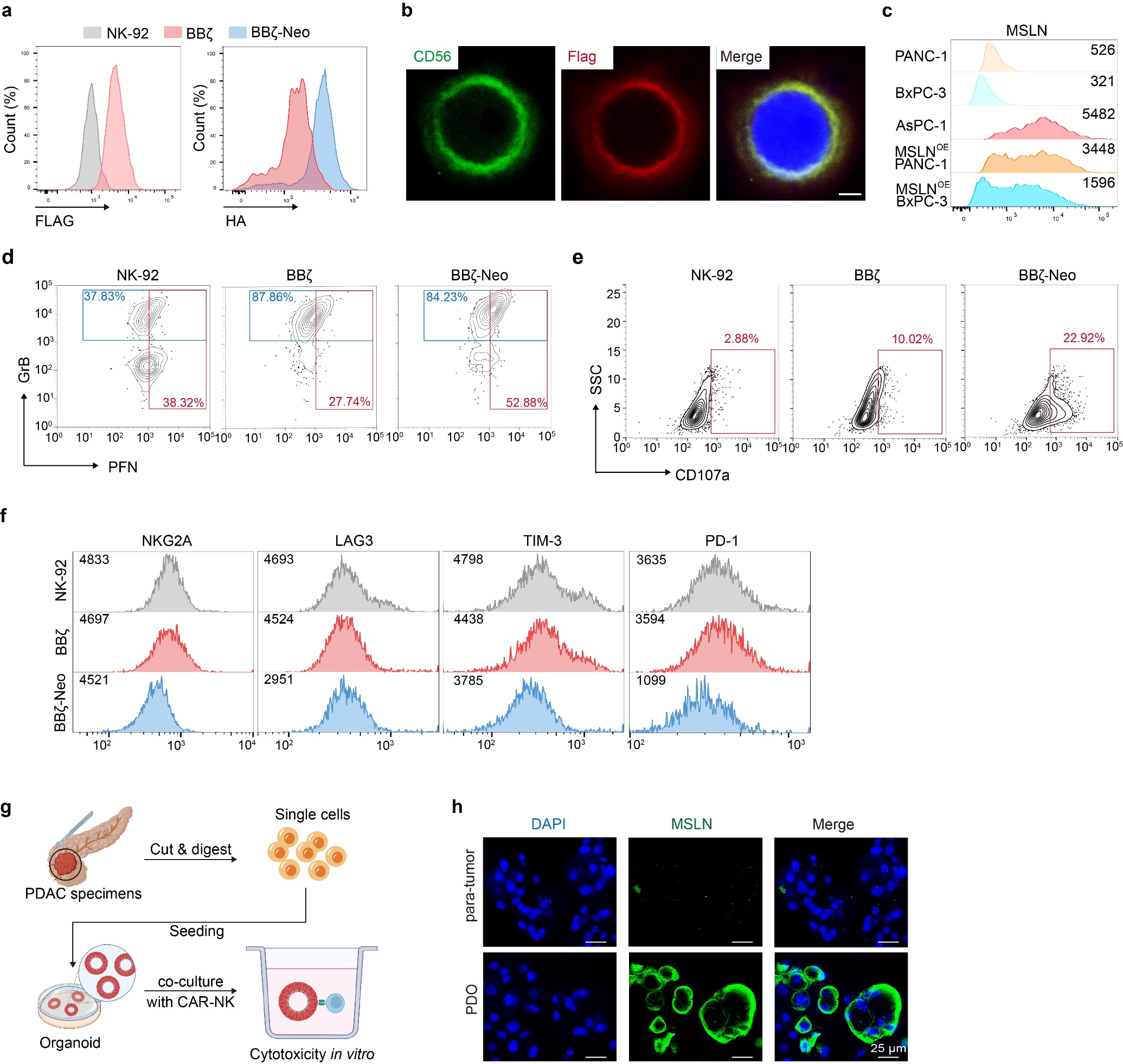


**Supplementary Fig. 3** | **Neo-2/15 enhances the cytotoxicity of CAR-NK cells *in vitro*. a**, The expression of Flag-tagged CAR and HA-tagged Neo-2/15 in NK-92 (gray), BBζ (red), and BBζ-Neo (blue) was determined by flow cytometry. **b**, The expression of Flag-tagged CAR (red) in BBζ was determined by IF. Scale bar=2 μm. **c**, The expression of MSLN in PANC-1, BxPC-3, AsPC-3, MSLN^OE^ PANC-1 and MSLN^OE^ BxPC-3 was determined by flow cytometry. **d-e**, Representative flow charts of the expression of PFN and GrB (**d**) and CD107a (**e**) in NK-92, BBζ, or BBζ-Neo after being co-cultured with AsPC-1 cells for 4 hours. **f**, Expression of NKG2A, LAG3, TIM-3, or PD-1 in NK-92, BBζ, and BBζ-Neo after co-culture with AsPC-1 cells for 4 hours was detected by flow cytometry. Representative images are shown. **g**, Schematic representation of organoid establishment, culture, and co-culture with CAR-NK cells. **h,** Representative images of MSLN expression in PDO by IF.


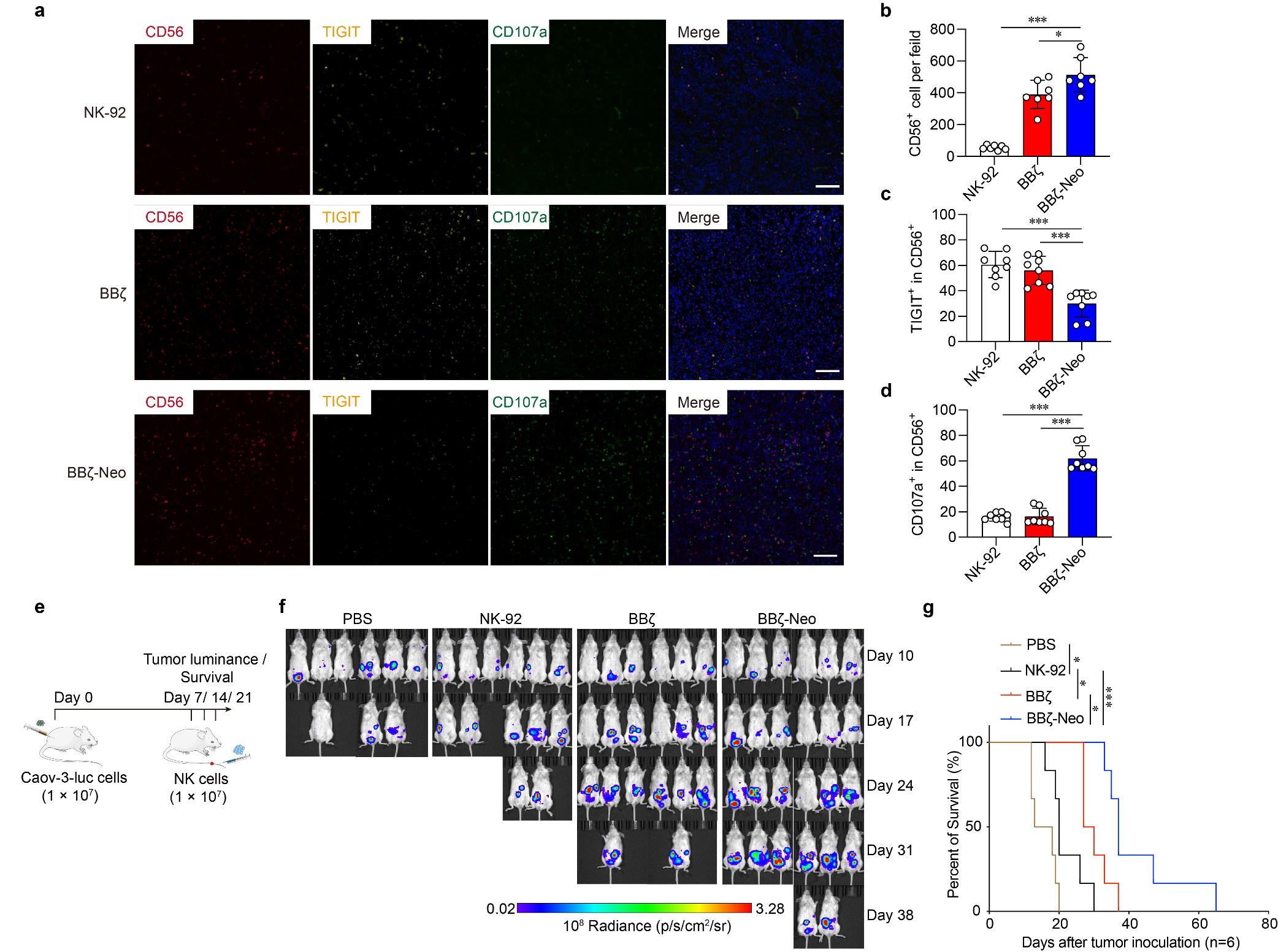


**Supplementary Fig.4** | **Autocrine Neo-2/15 strengthens the antitumor efficacy of CAR-NK cells.** **a**, The expression of CD56, TIGIT and CD107a of NK cells in tumor tissue after NK-92, BBζ, and BBζ-Neo treatment detected by multicolor IF. CD56 (red), TIGIT (orange), CD107a (green), DAPI (blue). Scale bar = 100 μm. **b-d**, Proportion of CD56^+^ cells (**b**), TIGIT^+^CD56^+^ cells (**c**), and CD107a^+^CD56^+^ cells (**d**) in the tumor tissue after NK-92, BBζ and BBζ-Neo treatment. Data are presented as mean ± SD (**P* < 0.05, ****P* < 0.001). **e**, Schematic outline of the development and evaluation of the ovarian cancer mouse model. **f**, Tumor burden was monitored weekly by measuring luminescence using IVIS imaging. **g**, Kaplan-Meier curve representing the percent survival of tumor-bearing mice treated with PBS, NK-92, BBζ, or BBζ-Neo. Statistics: two-tailed log-rank test. Data are presented as mean ± SD (**P <*0.05, ****P <*0.001, n=6).


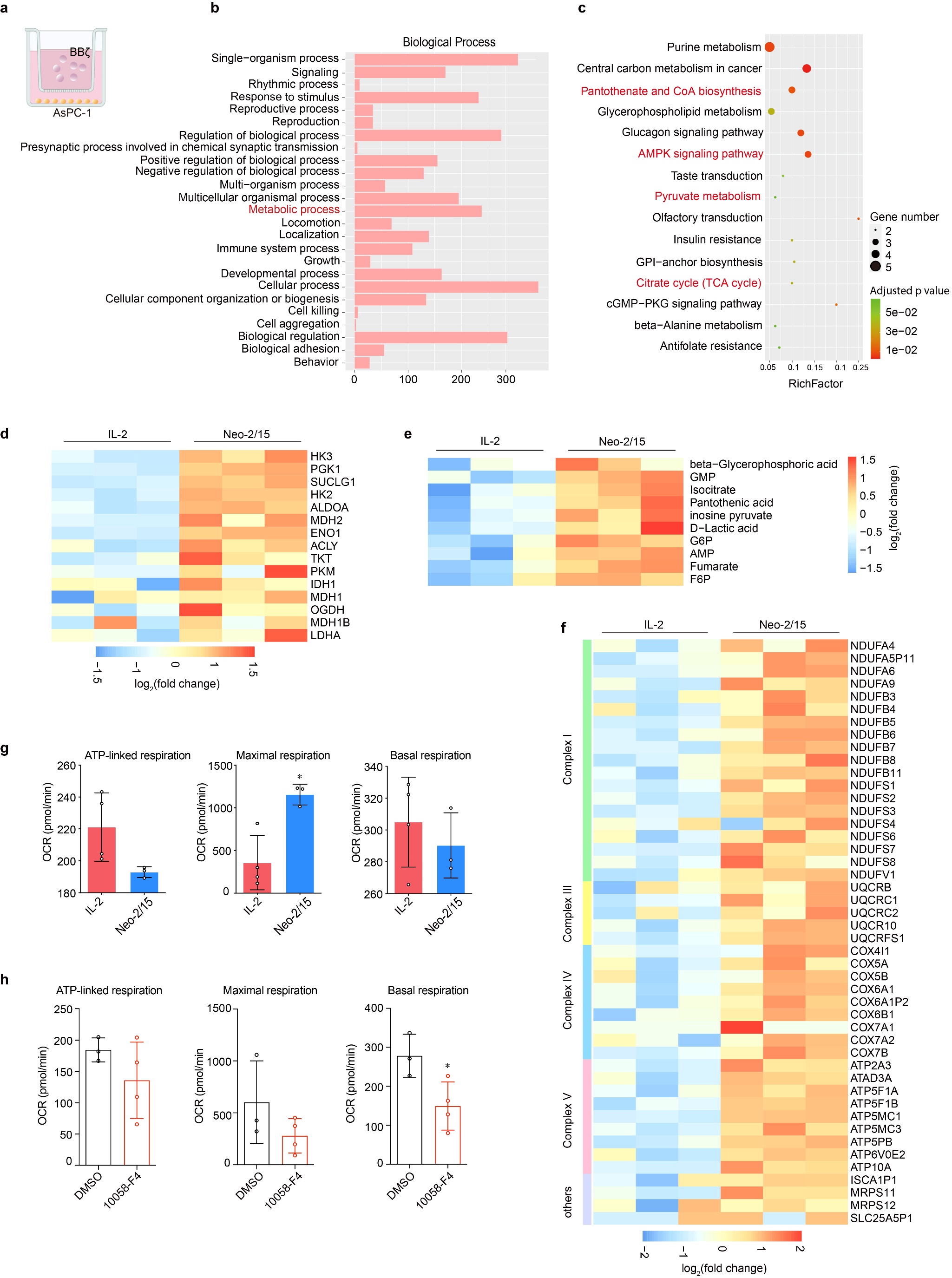


**Supplementary Fig.5** | **Neo-2/15 promotes ATP generation and OXPHOS of CAR-NK cells.** **a**, Co-culture scheme of CAR-NK cells with tumor cells. Specifically, AsPC-1 cells were cultured on the bottom of the 24-well plate, IL-2 or Neo-2/15 stimulated BBζ were added to a 40 μm transwell chamber. **b**, Transcriptome analysis for the identification of pathways in BBζ stimulated with Neo-2/15 versus IL-2 in co-culture with AsPC-1 cells. Pathways involved in the metabolic processes are highlighted in red. **c**, Metabolomics analysis for the enriched metabolite sets in BBζ stimulated with Neo-2/15 versus IL-2. Interested pathways are highlighted. **d-f**, Heatmaps of enzymes (**d**), metabolites (**e**), and mitochondrial respiratory chain complex (**f**) altered in BBζ stimulated with Neo-2/15 or IL-2 by log2-fold change in transcriptomic or metabolomics data. **g-h**, IL-2 or Neo-2/15 stimulated BBζ co-cultured with AsPC-1 cells for 4 hours (**g**), or Neo-2/15 stimulated BBζ pretreated with or without 10058-F4 (60 μM) overnight and co-cultured with AsPC-1 cells (**h**). BBζ were then collected and evaluated for OCR. Quantifications ATP-linked respiration, maximal respiration and basal respiration of BBζ are showed. Data are presented as mean ± SD (**P <*0.05).


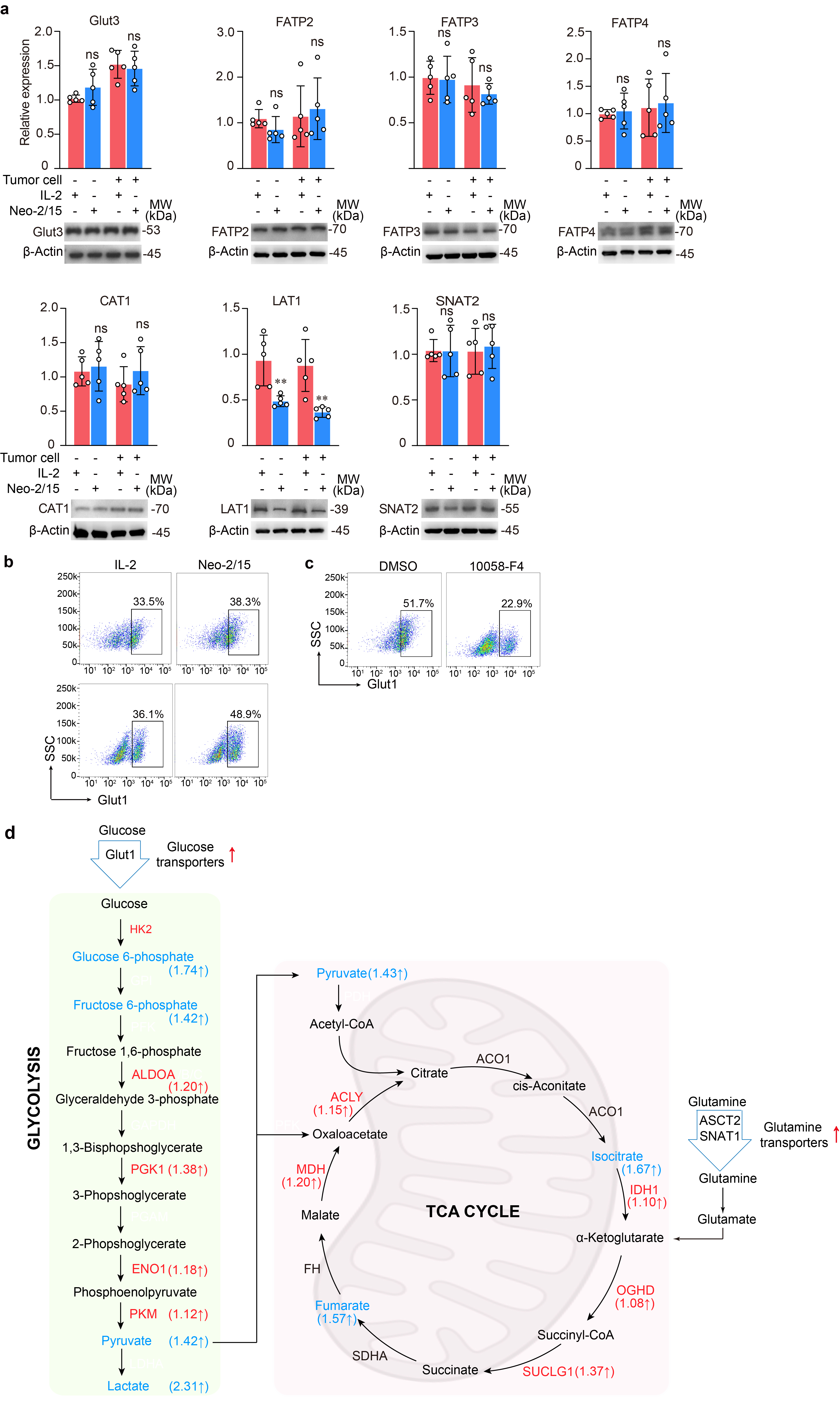


**Supplementary Fig.6** | **Neo-2/15 upregulates nutrient transporters of CAR-NK cells. a**, Expression of glucose and glutamine transporters was assessed in BBζ stimulated with Neo-2/15 or IL-2 and co-cultured with or without AsPC-1 cells for 4 hours. Data are presented as mean ± SD (ns, not significant, ***P* < 0.01, n=5). **b**, Expression of Glut1 was analyzed in BBζ stimulated with either IL-2 or Neo-2/15, co-cultured with or without AsPC-1 cells for 4 hours. **c**, Glut1 expression was assessed in Neo-2/15 expanded BBζ pretreated with or without 10058-F4 (60 μM) following co-cultured with AsPC-1 cells for 4 hours. **d**, A summary of altered enzymes, metabolites, and material transporters in BBζ under Neo-2/15 stimulation. blue: metabolites; red: enzymes; upward arrow: upregulation. Blue and red numbers respectively show fold changes in metabolites or transcript read counts compared to the IL-2 group.


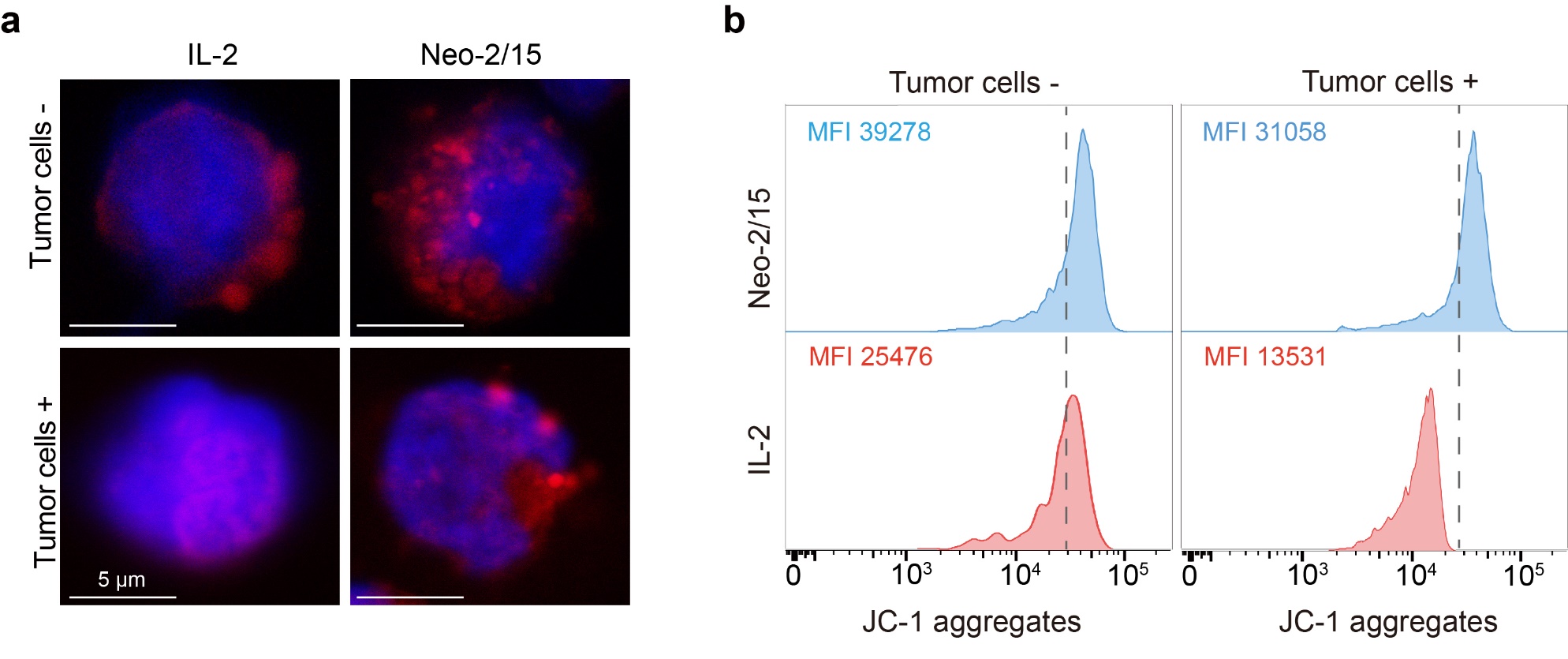


**Supplementary Fig.7** | **Neo-2/15 sustains mitochondrial homeostasis and apoptosis resistance of CAR-NK. a**, Representative confocal laser scanning microscopy images showing stained mitochondria (MitoTracker; red) and nucleus (DAPI; blue) in IL-2 or Neo-2/15 stimulated BBζ co-cultured with or without AsPC-1 cells for 4 hours at E:T ratio of 1:1. **b,** Representation of flow cytometry images of JC-1 aggregation in IL-2 or Neo-2/15 stimulated BBζ co-cultured with or without AsPC-1 cells for 4 hours at E:T ratio of 1:1 and stained with JC-1.


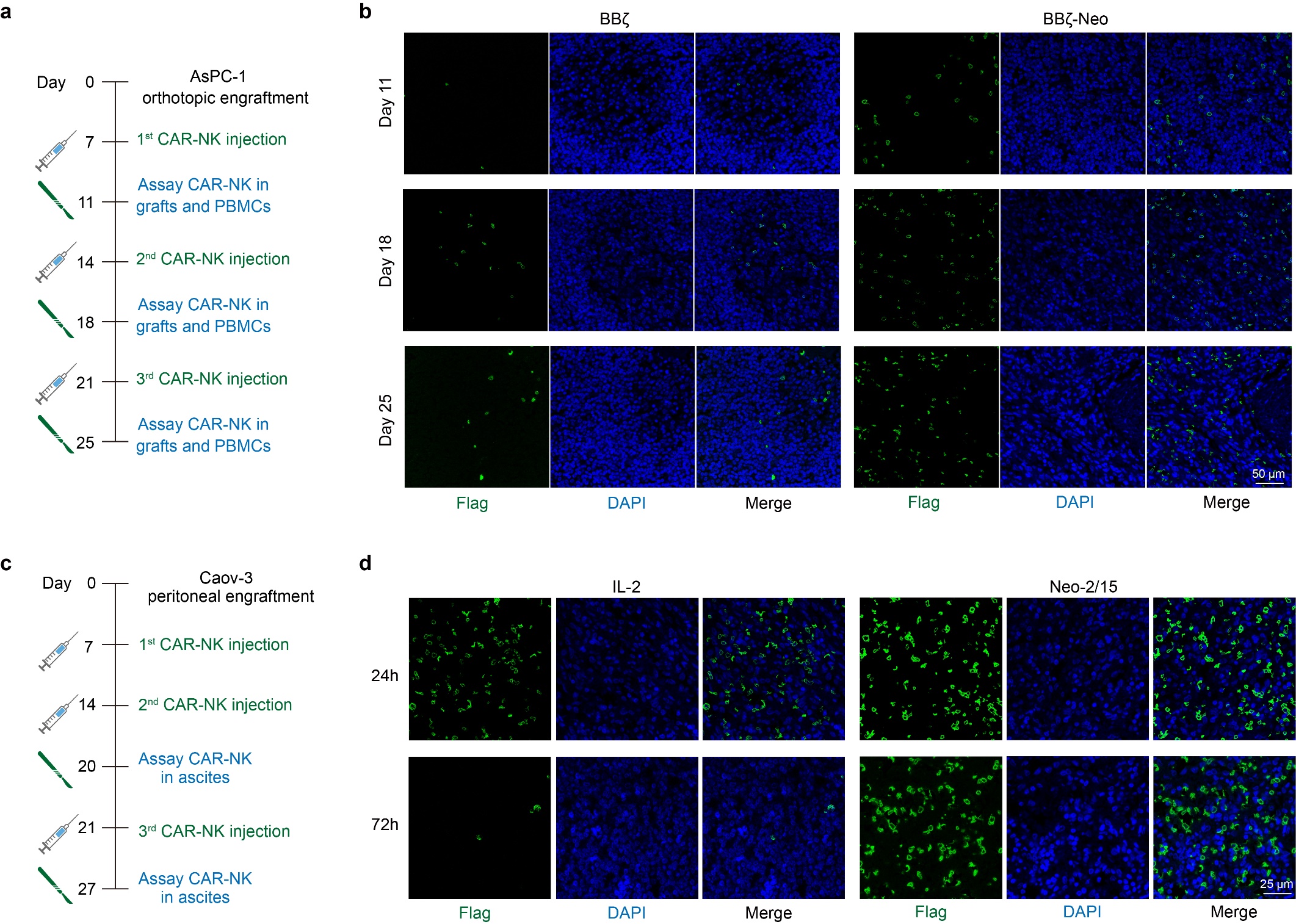


**Supplementary Fig.8** | **Neo-2/15 enhances survival of CAR-NK cells within TME.** **a**, Schematic of the treatment regimen for orthotopically engrafted pancreatic cancer using CAR-NK cells, including subsequent analysis of NK cells within the tumor tissues. **b**, Representative IF images showing the survival of CAR-NK cells within tumor from the orthotopic pancreatic cancer model. **c**, Schematic representation of the treatment of ovarian cancer xenograft model with CAR-NK cells and analysis of CAR-NK cells in the ascites. **d**, Representative IF images of intratumoral CAR-NK cells expanded by IL-2 or Neo-2/15 and pretreated with mitomycin C (10 μg/mL) as described in **Fig. 5d**.


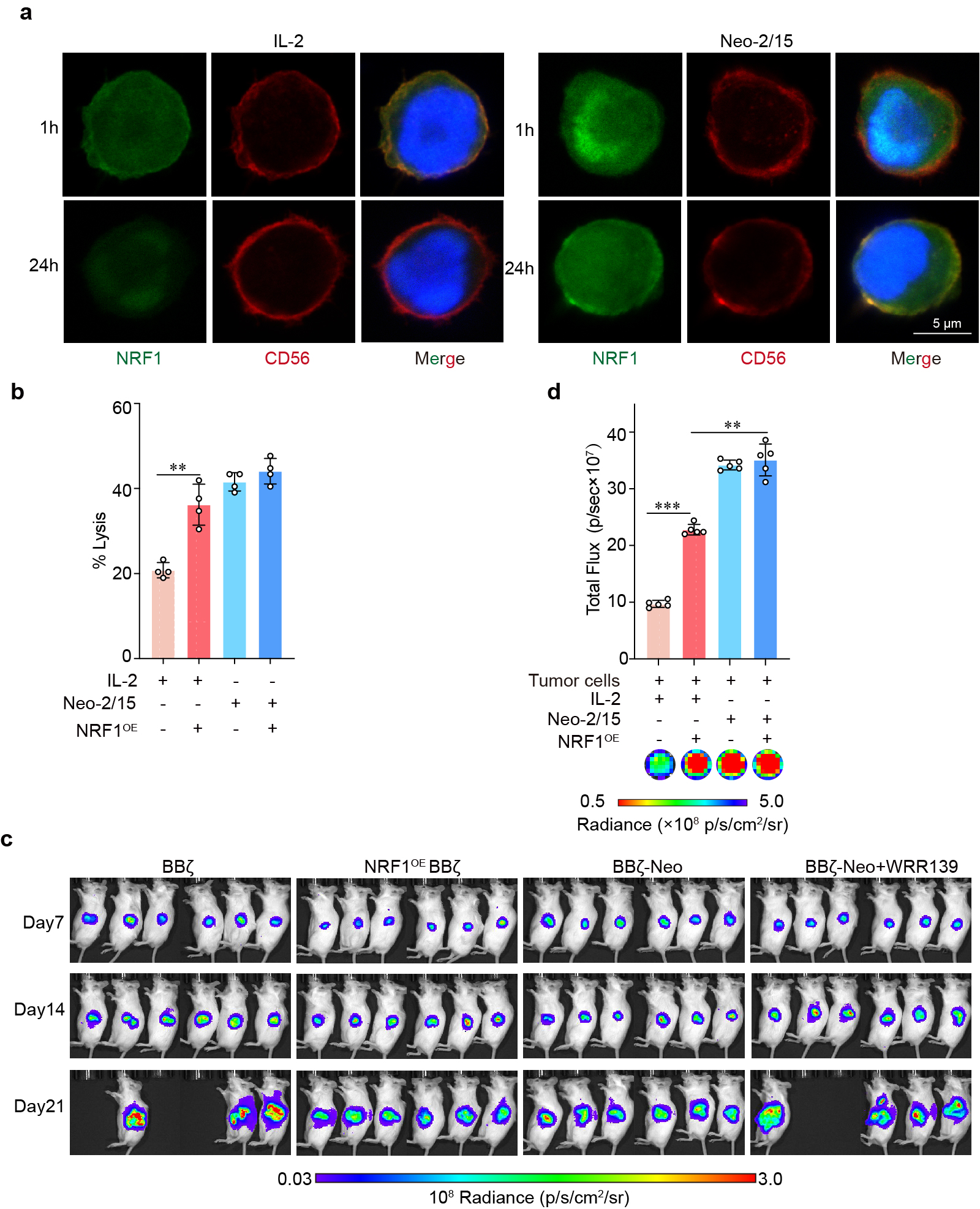


**Supplementary Fig. 9** | **NRF1 overexpression enhances antitumor efficacy of CAR-NK cells.** **a**, BBζ expanded with IL-2 or Neo-2/15 were harvested from AsPC-1 grafts at indicated timepoints post-injection. NRF1 (green) expression in BBζ (CD56, red) was measured by IF staining. **b**, IL-2 or Neo-2/15 stimulated BBζ or NRF1^OE^ BBζ were co-cultured with AsPC-1 cells for 4 hours at the E:T ratio of 1:1. The cytotoxicity was evaluated. Data are presented as mean ± SD (***P* < 0.01, n=4). **c**, Tumor-bearing mice were established and then treated with 1×10^7^ BBζ, NRF1^OE^ BBζ, BBζ-Neo, or BBζ-Neo with 2 μg/mL WRR139 administered intraperitoneally on days 7, 14, and 21 post-tumor inoculation. Tumor burden was monitored weekly by measuring luminescence using IVIS imaging. **d**, IL-2 or Neo-2/15-stimulated BBζ or NRF1^OE^ BBζ co-cultured with AsPC-1 cells in transwell chamber for 4 hours at the E:T ratio of 1:1. The ATP generation was evaluated. Data are presented as mean ± SD (***P* < 0.01, ****P* < 0.001, n=5).


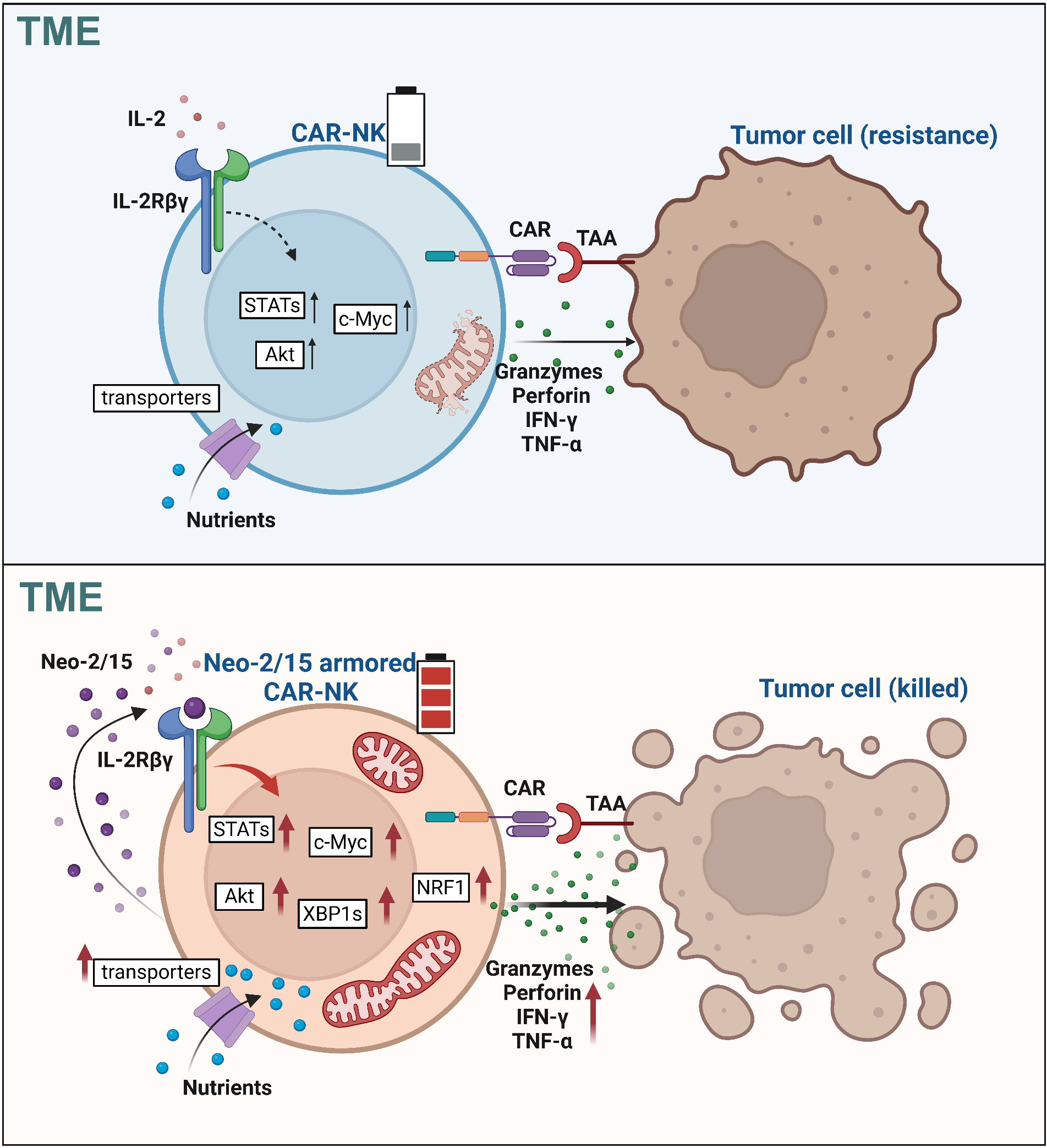


**Supplementary Fig. 10** | **The graphical summary highlights the main findings of this study.** Upper panel: Natural IL-2 stimulated CAR-NK cells encounter resistance from tumor cells, highlighting the limited persistence, infiltration, and increased exhaustion due to inadequate nutrient and cytokine support. Lower panel: Neo-2/15-armored CAR-NK cells exhibit enhanced metabolic adaptation and mitochondrial fitness via activation of c-Myc/NRF1 signaling pathways. The engineered cells demonstrate increased cytotoxicity accompanied by increased levels of perforin, granzymes, and inflammatory cytokines like IFN-γ and TNF-α, leading to effective tumor cell eradication. The overexpression of NRF1 further supports enhanced ATP production, contributing to prolonged persistence and reduced exhaustion of CAR-NK cells within the TME (created using BioRender).

**Supplementary Table 1. The amino acid sequence of αMSLN-CAR and Neo-2/15**

| **Name** | **Amino acid sequence** |
| --- | --- |
| anti-MSLN CAR | MALPVTALLLPLALLLHAARPDYKDDDDKGGGGSDVVMTQTPASVSEPVGGTVTIKCQASQRISSYLSWYQQKPGQRPKLLIFGASTLASGVPSRFKGSGSGTEYTLTISDLECADAATYYCQSYAYFDSNNWHAFGGGTEVVVDYKDDDDKDYKDDDDKQQQLEESGGGLVKPEGSLTLTCKASGFDLGFYFYACWVRQAPGKGLEWIACIYTAGSGSTYYASWAKGRFTISKASSTTVTLQMTSLAAADTATYFCARSTANTRSTYYLNLWGPGTLVTVSSTTTPAPRPPTPAPTIASQPLSLRPEACRPAAGGAVHTRGLDFACDIYIWAPLAGTCGVLLLSLVITLYCKRGRKKLLYIFKQPFMRPVQTTQEEDGCSCRFPEEEEGGCELRVKFSRSADAPAYQQGQNQLYNELNLGRREEYDVLDKRRGRDPEMGGKPRRKNPQEGLYNELQKDKMAEAYSEIGMKGERRRGKGHDGLYQGLSTATKDTYDALHMQALPPREFGSG |
| Neo-2/15 | MALWMRLLPLLALLALWGPDPAAAYPYDVPDYAGSHMPKKKIQLHAEHALYDALMILNIVKTNSPPAEEKLEDYAFNFELILEEIARLFESGDQKDEAEKAKRMKEWMKRIKTTASEDEQEEMANAIITILQSWIFS |
